# Inhibition of Plasmepsin V Activity Demonstrates Its Essential Role in Protein Export, PfEMP1 Display, and Survival of Malaria Parasites

**DOI:** 10.1371/journal.pbio.1001897

**Published:** 2014-07-01

**Authors:** Brad E. Sleebs, Sash Lopaticki, Danushka S. Marapana, Matthew T. O'Neill, Pravin Rajasekaran, Michelle Gazdik, Svenja Günther, Lachlan W. Whitehead, Kym N. Lowes, Lea Barfod, Lars Hviid, Philip J. Shaw, Anthony N. Hodder, Brian J. Smith, Alan F. Cowman, Justin A. Boddey

**Affiliations:** 1 The Walter and Eliza Hall Institute of Medical Research, Parkville, Victoria, Australia; 2 Department of Medical Biology, The University of Melbourne, Parkville, Victoria, Australia; 3 University of Copenhagen and Copenhagen University Hospital (Rigshospitalet), Copenhagen, Denmark; 4 National Center for Genetic Engineering and Biotechnology (BIOTEC), Pathum Thani, Thailand; 5 Department of Chemistry, La Trobe University, Victoria, Australia; University of Georgia, United States of America

## Abstract

The malaria parasite *Plasmodium falciparum* exports several hundred proteins into the infected erythrocyte that are involved in cellular remodeling and severe virulence. The export mechanism involves the *Plasmodium* export element (PEXEL), which is a cleavage site for the parasite protease, Plasmepsin V (PMV). The *PMV* gene is refractory to deletion, suggesting it is essential, but definitive proof is lacking. Here, we generated a PEXEL-mimetic inhibitor that potently blocks the activity of PMV isolated from *P. falciparum* and *Plasmodium vivax*. Assessment of PMV activity in *P. falciparum* revealed PEXEL cleavage occurs cotranslationaly, similar to signal peptidase. Treatment of *P. falciparum*–infected erythrocytes with the inhibitor caused dose-dependent inhibition of PEXEL processing as well as protein export, including impaired display of the major virulence adhesin, PfEMP1, on the erythrocyte surface, and cytoadherence. The inhibitor killed parasites at the trophozoite stage and knockdown of PMV enhanced sensitivity to the inhibitor, while overexpression of PMV increased resistance. This provides the first direct evidence that PMV activity is essential for protein export in *Plasmodium* spp. and for parasite survival in human erythrocytes and validates PMV as an antimalarial drug target.

## Abstract 2

A small molecule inhibitor of the malarial protease Plasmepsin V impairs protein export and cellular remodeling, reducing parasite survival in human erythrocytes.

## Author Summary

To survive within human red blood cells, malaria parasites must export a catalog of proteins that remodel the host cell and its surface. This enables parasites to acquire nutrients from outside the cell and to modify the cell surface in order to evade host defenses. Protein export involves proteolytic cleavage of the *Plasmodium* Export Element (PEXEL) by the aspartyl protease Plasmepsin V. We report here the development of a small molecule inhibitor that closely mimics the natural PEXEL substrate and blocks the activity of Plasmepsin V from the malarial parasites *Plasmodium falciparum* and *Plasmodium vivax*. The inhibitor impairs export and cellular remodeling and kills *P. falciparum* at the ring-trophozoite transition, providing direct evidence that Plasmepsin V activity is essential for export of PEXEL proteins and parasite survival within the host. These findings validate Plasmepsin V as a highly conserved antimalarial drug target.

## Introduction

Each year malaria parasites cause several hundred million infections and over 650,000 deaths [1]. *Plasmodium falciparum* causes the most lethal malaria and is endemic in Africa [2]. *Plasmodium vivax* causes most malarial deaths outside Africa and is associated with liver-stage hypnozoites [3]. Although chloroquine and artemisinin have been effective antimalarials, their decreasing efficacy [4],[5] emphasizes the need for therapies against novel targets shared by both *Plasmodium* spp.

Malaria parasites develop in erythrocytes within a parasitophorous vacuole and export over 450 proteins to the cell (reviewed in [6],[7]). Export utilizes an N-terminal motif called the *Plasmodium* export element (PEXEL; RxLxE/Q/D) [8] or Vacuolar transport signal (VTS) [9]. Exported proteins are cleaved in the PEXEL after Leu (RxL↓) in the endoplasmic reticulum (ER) [10], which requires the conserved Arg and Leu residues [11]. PEXEL cleavage is performed by the aspartyl protease Plasmepsin V (PMV) [12],[13]. PEXEL-containing proteins and PMV are conserved in all *Plasmodium* spp. [8],[14]–[16]. Repeated attempts to disrupt the *PMV* gene have failed, suggesting it is essential [12],[13],[16], but direct and decisive proof is still lacking. A functional survey of *P. falciparum* exported proteins indicated that 25% or more are essential for parasite survival in human erythrocytes [17]. The current *P. falciparum* PEXEL exportome is predicted to be 463 proteins [18]; thus, possibly 100 or more exported parasite proteins are required for development in erythrocytes.

Some exported proteins lack a PEXEL, for example, skeleton binding protein 1 (SBP1) and the major virulence adhesin family known as *P. falciparum* erythrocyte membrane protein 1 (PfEMP1). PfEMP1 is expressed on the erythrocyte surface and mediates cytoadherence to microvascular endothelia, causing severe malaria [19]. PfEMP1 is thought not to be cleaved by PMV [18], but its transport to, and expression on, the erythrocyte surface requires exported PEXEL and PEXEL-negative proteins (reviewed in [7],[20]).

Aspartyl proteases can be inhibited by transition-state isosteres in which the scissile bond is replaced by a noncleavable moiety. Examples include statine (Sta)-containing inhibitors and several are now in clinical use [21].

Here, we developed a transition-state inhibitor that potently blocks PMV from *P. falciparum* and *P. vivax*. The inhibitor demonstrates that PMV activity is essential for protein export, PfEMP1 surface display, cytoadherence, and parasite survival in human erythrocytes.

## Results

### PMV Is Highly Conserved in *Plasmodium*

**Figure 1:**
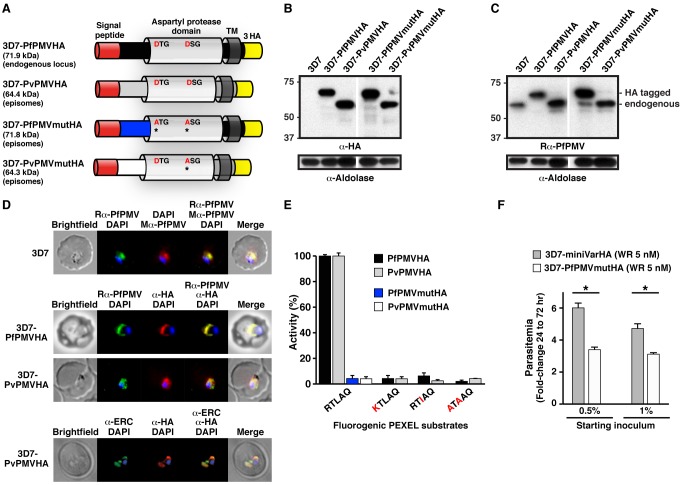
PMV conservation and expression. (A) Structure and size of PMVHA proteins used in this study. Catalytic dyad residues DTG/DSG are shown including Asp to Ala mutations* in red. TM, transmembrane domain. (B) Immunoblot of infected erythrocytes with α-HA antibodies shows expression of PMVHA proteins in *P. falciparum*. Sizes indicate that the signal peptides were removed (PfPMVHA, 69.1 kDa; PvPMVHA, 60.9 kDa). (C) Immunoblotting of infected erythrocytes with rabbit α-PfPMV antibodies (Rα-PfPMV) validates they are specific for PfPMV. Endogenous PfPMV is the lower band (lanes 1, 3, 4, 5), and the larger band corresponds to 3× HA-tagged PfPMV (lanes 2, 4). Aldolase is a loading control in (B) and (C) and shows slight overloading of some lanes compared to others. (D, Top) Immunofluorescence micrographs show rabbit α-PfPMV antibodies (Rα-PfPMV, green) label PfPMV in the ER. Colocalizations were performed with mouse α-PfPMV antibodies (Mα-PfPMV, red), shown previously to label PMV in the ER [16]. (Middle) α-HA antibodies (red) label PfPMVHA (Top) and PvPMVHA (Bottom) in the parasite ER. (Bottom) α-HA antibodies (red) label PvPMVHA in the ER, as shown by clocalization with ERC (green). (E) Immunopurified PfPMVHA and PvPMVHA cleave KAHRP peptides containing the PEXEL sequence RTLAQ but not peptides containing point mutations R>K, L>I, or RL>A. Pf and Pv PMVmutHA proteins with catalytic D>A mutations did not cleave the KAHRP RTLAQ peptide. (F) Overexpression of PfPMVmutHA from episomes in *P. falciparum* 3D7 impairs growth relative to expression of a similar episomal construct encoding a mini PfEMP1HA reporter (miniVarHA). Parasites expressing episomes were selected on 5 nM WR99210 (WR). Two starting inocula were used in triplicate wells, and parasitaemia was determined at 72 h. *p<.0001 (*t* test). Data are mean ± SEM from duplicate experiments.

The *PMV* gene is present in all *Plasmodium* spp.; however, only the *P. falciparum* enzyme (PfPMV; Pf3D7_1323500) has been characterized. A multiple alignment of PfPMV with putative *P. vivax* PMV (PvPMV; PVX_116695) indicated that they share 82.2% similarity, 54.7% identity (Figure S1). Both proteins are predicted to contain a signal peptide, an aspartyl protease domain with DTG and DSG residues defining the catalytic dyad, and a C-terminal transmembrane domain (Figure 1A). Due to four insertions, PfPMVHA is predicted to be approximately 7.5 kDa larger than PvPMVHA (Figure 1A); however, following signal peptide removal, PfPMVHA is predicted to be 8 kDa larger than PvPMVHA.

To determine whether PvPMV is an ortholog of PfPMV, we expressed it in *P. falciparum* fused to 3× hemagglutinin (HA) tags (Figure 1A). As a positive control, we expressed PfPMV fused to 3× HA tags (Figure 1A) [13]. Expression of PfPMVHA and PvPMVHA was confirmed by immunoblot using anti-HA antibodies (Figure 1B). PvPMVHA was ∼8 kDa smaller than PfPMVHA, as predicted (Figure 1B).

PfPMV was previously localized to the ER using a mouse anti-PfPMV antibody that colocalizes with BiP [16] and ERC [13]. To further study PMV, we developed a rabbit antibody that was specific for PfPMV (Figure 1C, compare lanes 1 and 2) and colocalizes with the ER signal from the mouse PfPMV antibody (Figure 1D, top) but does not cross-react with PvPMVHA (Figure S2A). Using anti-HA antibodies, a strong perinuclear signal was observed in parasites expressing PvPMVHA or PfPMVHA (Figure 1D, middle panels, red). Both proteins colocalized with rabbit anti-PfPMV antibodies, indicating the location was the ER (Figure 1D). PvPMVHA also colocalized with ERC (Figure 1D, bottom), as shown previously for PfPMVHA [13].

To investigate PvPMVHA activity, we affinity purified it, as well as the PfPMVHA control, using anti-HA agarose, as previously described [13],[18]. Immunoblot with anti-HA and anti-PfPMV antibodies showed that the purified proteins were species-specific (Figure S2A). The proteins were incubated with a fluorogenic peptide of nine amino acids that contained the PEXEL sequence from knob-associated histidine-rich protein (KAHRP), and efficient processing was observed by both enzymes (Figure 1E). Cleavage of KAHRP by PfPMVHA was previously shown to occur after Leu (RTL↓) by mass spectrometry [13], and this position was also confirmed for PvPMVHA (Figure S2B–E). *Km* values of 9.7 (±3.0) and 11.7 (±1.8) µM were calculated for PvPMVHA and PfPMVHA, respectively (Figure S2F). In contrast, no processing was observed when the PEXEL Arg and Leu residues were mutated to Ala (ATAAQ), consistent with the substrate specificity of PfPMV (Figure 1E). To verify that processing was due to HA-tagged PMV rather than other co-precipitated proteases, we expressed in *P. falciparum* a mutant of PvPMVHA or PfPMVHA [13], where one or both catalytic Asp residues were mutated to Ala (see Figure 1A,B). Following affinity purification, the mutant enzymes were incubated with KAHRP PEXEL peptides, but no processing was observed (Figure 1E; blue, white), confirming that the PEXEL-dependent cleavage activity observed for each protease was attributable only to HA-tagged PMV.

The substrate specificity of PfPMV is restricted, such that even minor changes to the PEXEL sequence markedly reduces cleavage efficiency—that is, Arg to Lys (R>K) or Leu to Ile (L>I) [18]. We assessed whether PvPMVHA shares this feature. Indeed, PvPMVHA poorly cleaved peptides possessing R>K or L>I mutations (Figure 1E; KTLAQ, RTIAQ). Collectively, these data show that PvPMV localizes to the ER and cleaves the PEXEL with the same restricted specificity as PfPMV.

While maintaining *P. falciparum* cultures overexpressing PfPMVmutHA, we noticed a delay in growth, suggesting a possible dominant negative phenotype, which has been reported previously with a different PfPMV catalytic mutant [12]. A flow cytometry-based growth assay revealed that parasites expressing PfPMVmutHA with WR99210 selection grew to a parasitemia 2.6-fold less than parasites expressing a similar episomal construct, encoding a mini PfEMP1 reporter fused to 3× HA tags (miniVarHA) with WR99210 selection (*p*<.0001; Figure 1F). This demonstrated that overexpression of inactive enzyme conveyed a growth disadvantage, providing evidence that PMV is important for parasite survival. Analysis of PMV protein levels in parasites overexpressing the PMVHA transgenes indicated that PvPMVHA and PvPMVmutHA had no effect on endogenous PfPMV levels; however, overexpression of inactive PfPMVmutHA caused a clear decrease in expression of the endogenous enzyme (Figure 1C, compare lanes 1 and 4, and Aldolase loading controls), indicating a negative feedback mechanism occurs in these parasites.

### Rational Design of a PMV Inhibitor

**Figure 2:**
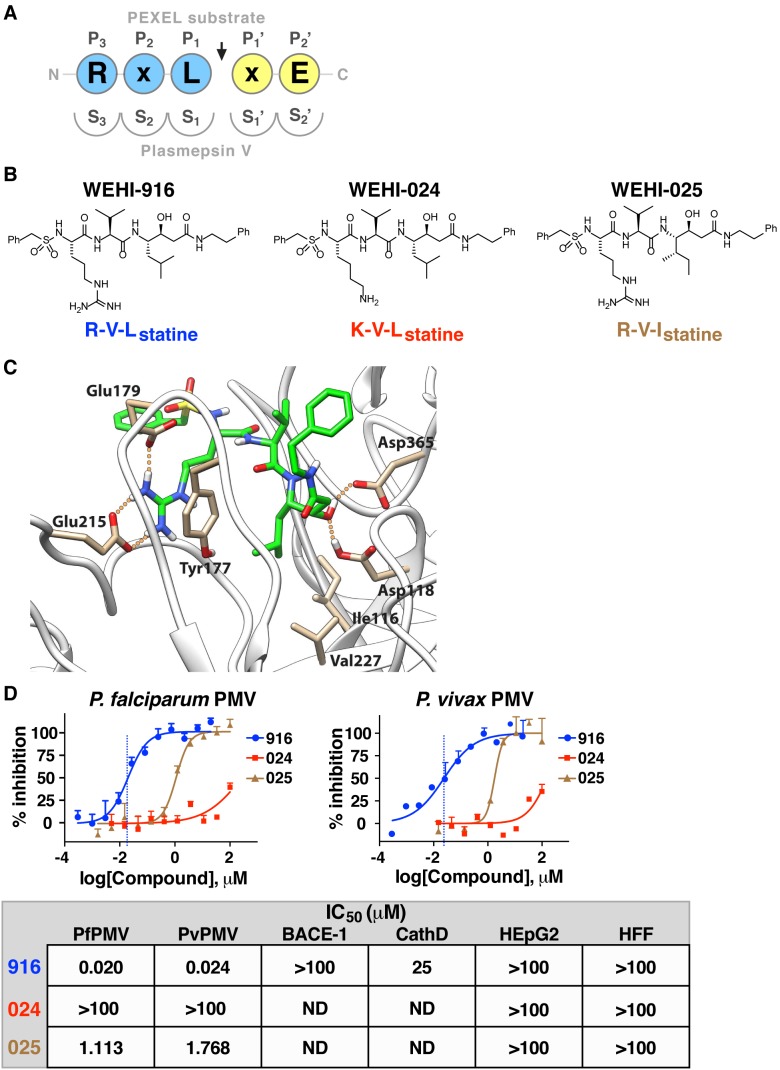
A PEXEL-mimetic inhibitor of PMV. (A) Nomenclature for each residue in the PEXEL substrate (circles) and each pocket of the PMV active site (semicircles) with respect to the cleavage site (arrow). (B) Compound structures in this study include PMV inhibitor WEHI-916 and control analogs WEHI-024 and WEHI-025. (C) Structural model of PfPMV bound to WEHI-916. Residues forming the S_3_ site, Try177, Glu179, and Glu215, form interactions with the guanidine side chain of WEHI-916. The Leu side chain of WEHI-916 packs tightly against the side-chain groups of Ile116, Tyr177, and Val227 in the S_1_ site. The statine hydroxyl forms hydrogen bonds with the two catalytic aspartate residues Asp118 and Asp365. (D) Inhibition of PfPMV and PvPMV by WEHI-916 (blue) and weak activities of WEHI-024 (red) and WEHI-025 (brown). The grey box summarizes compound activity against BACE-1 and Cathepsin D (CathD) and lack of toxicity against human HEpG2 cells and Human Foreskin Fibroblasts (HFF).

The conserved P_3_ Arg and P_1_ Leu residues in the PEXEL (see Figure 2A for a description of nomenclature) are crucial for PMV activity [12],[13]. We developed a homology model and designed compounds with a transition-state isostere that mimics the natural PEXEL substrate with the aim of inhibiting PMV. One mimetic, WEHI-916 (Figure 2B), consisted of Arg that could bind in the S_3_ pocket of PfPMV, Val that would position in the S_2_ pocket, and Leu-Statine (Leu-Sta), to engage the S_1_ pocket and inhibit both catalytic Asp residues of PMV (Figure 2C).

As control compounds, we synthesized analogs similar to 916 but that mimic noncleavable PEXEL mutant substrates, with the aim that they would be poor PMV inhibitors; the first replaced the P_3_ Arg with Lys (R>K; WEHI-024) and the second replaced the P_1_ Leu with Ile (L>I; WEHI-025; Figure 2B). These analogs were designed on the basis that mutations of the conserved PEXEL residues R>K or L>I almost completely inhibit cleavage by PMV (Figure 1E and [18]) and should therefore have lower affinity for PMV.

Each compound was incubated with PfPMVHA in the presence of KAHRP PEXEL peptides. WEHI-916 (henceforth 916) potently inhibited PEXEL cleavage by PfPMVHA with a 50% inhibitory concentration (IC_50_) of 20 nM (Figure 2D). In contrast, WEHI-024 and WEHI-025 (henceforth 024 and 025, respectively) had weak activity (IC_50_>100 µM and 1.11 µM, respectively; Figure 2D). 916 inhibited PvPMVHA with an IC_50_ of 24 nM, whereas 024 and 025 again had weak activity (Figure 2D).

To investigate potential off-target activity against human aspartyl proteases, the compounds were assessed against beta-secretase (BACE-1), for which PMV has distant relatedness [12], Cathepsin D, and two human cell lines; the compounds displayed poor activity (IC_50_>100 µM for BACE-1; 25 µM for Cathepsin D) and had negligible toxicity against human HEpG2 and fibroblast cell lines (Figure 2D). Collectively, this demonstrated that 916 potently inhibited PMV and had low off-target activity against BACE-1 and Cathepsin D, whereas the closely related analogs 024 and 025 were poorly active.

### WEHI-916 Inhibits PMV in *P. falciparum*–Infected Erythrocytes

**Figure 3:**
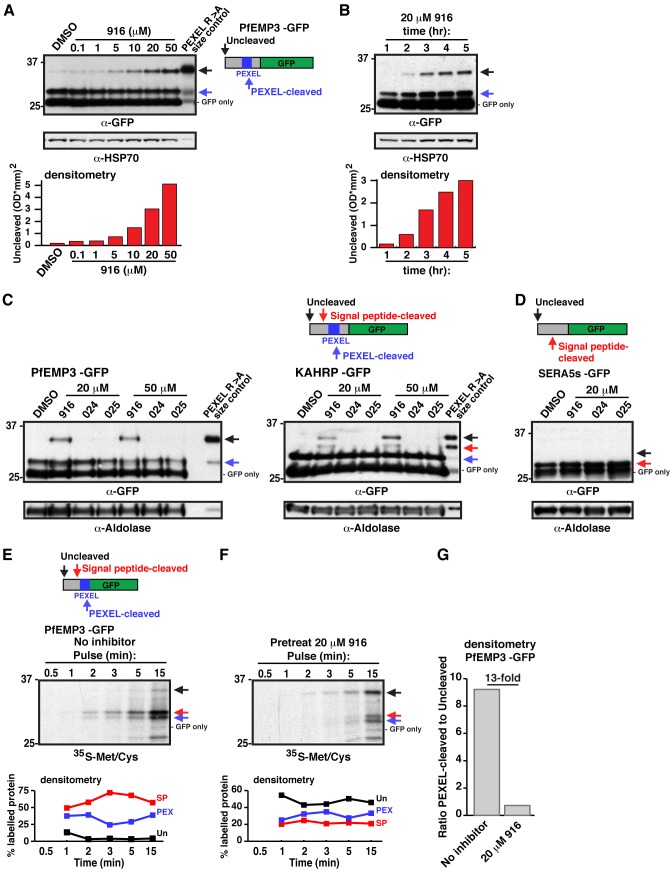
WEHI-916 blocks PEXEL cleavage in *P. falciparum*. (A) Immunoblotting with α-GFP antibodies shows dose-dependent inhibition of PEXEL cleavage of PfEMP3-GFP in parasites after 5 h 916 treatment at the indicated concentrations. Uncleaved protein (black arrow), PEXEL-cleaved protein (blue arrow), and degraded chimera in food vacuole (GFP only) are labeled. PfEMP3-GFP R>A PEXEL mutant is shown as a size control. (B) Immunoblot shows time-dependent inhibition of PEXEL cleavage in parasites. HSP70 is a loading control in (A) and (B) and densitometry of the uncleaved band in each lane is shown below the blots in (A) and (B). (C) Immunoblot shows 916 treatment (20 and 50 µM for 5 h) causes accumulation of uncleaved PfEMP3-GFP and KAHRP-GFP (black arrow), but 024 and 025 have no effect. R>A PEXEL mutant size controls are shown. Signal peptide-cleaved species of KAHRP-GFP, but not PfEMP3-GFP, can be seen (red arrow). (D) Immunoblotting shows no defect (black arrow indicates the predicted size of uncleaved protein; ∼30 kDa) in signal peptide cleavage (red arrow; 27 kDa) of SERA5s-GFP, which lacks a PEXEL, following treatment with 916, 024, or 025 (20 µM for 5 h). Aldolase was a loading control in (C) and (D). (E) ^35^S-Methionine/Cysteine labeling of PfEMP3-GFP in parasites reveals the rapid rate of translation, ER import, and N-terminal processing in *P. falciparum*. Uncleaved (black arrow), signal peptide-cleaved (red arrow), PEXEL-cleaved (blue arrow), and GFP-only (food vacuole) species are shown. Densitometry of each band per lane (colored traces) is shown. Uncleaved (Un, black), signal peptide-cleaved (SP, red), and PEXEL-cleaved (PEX, blue) bands are shown as percentage of total intensity per lane. Signal peptide-cleaved PfEMP3-GFP can be seen (red arrow, trace). (F) The experiment in (E) was performed after 916 treatment (20 µM for 5 h). Uncleaved protein was most abundant. Note low intensity of bands indicated by red and blue arrows and delay in their appearance compared to (E). (G) Densitometry showing the ratio of PEXEL-cleaved to -uncleaved PfEMP3-GFP with or without 916 treatment (determined using 15 min lanes in (E) and (F)).

To assess whether 916 could inhibit PMV in *P. falciparum*–infected erythrocytes, parasites expressing the PEXEL protein *P. falciparum* erythrocyte membrane protein 3 (PfEMP3) fused to green fluorescent protein (GFP) [13] were treated with increasing concentrations of inhibitor, and PEXEL processing was evaluated by immunoblot. A dose-dependent increase in unprocessed PfEMP3-GFP was observed (black arrow, Figure 3A), which was the same size as uncleaved PEXEL R>A mutant PfEMP3-GFP (Figure 3A) [13]. The level of PEXEL-cleaved protein (blue arrow, Figure 3A) did not quantitatively reflect the degree of PMV inhibition, as inhibitor was added well after PEXEL processing and export of PfEMP3-GFP had initiated. A GFP-only band, representing degraded chimera in the food vacuole, was also observed at ∼26 kDa (Figure 3A). Together, this demonstrated that PEXEL processing was impaired by 916 treatment and that engagement of PMV occurred in *P. falciparum*–infected erythrocytes.

To understand the timing required for PMV inhibition in *P. falciparum*, parasites were treated with 916 for 1–5 h, and cleavage was evaluated by immunoblot. No effect was seen after 1 h; however, uncleaved PfEMP3-GFP increased between 2 and 5 h (Figure 3B), indicating 916 accessed the parasite ER slowly. Inhibition of PfEMP3-GFP cleavage by 916 was rescued following culture in inhibitor-free medium, to approximately 50% after 2 h (Figure S3A), indicating that cleavage inhibition was reversible or that additional active PMV was synthesized during the experiment.

We next assessed whether the control analogs 024 and 025, which were poor inhibitors of PMV *in vitro*, had an effect on PEXEL cleavage in parasites. Although a dose-dependent effect was again observed with 916, analogs 024 and 025 had no effect on PEXEL processing of PfEMP3-GFP or KAHRP-GFP, even at 50 µM (Figure 3C). In the case of KAHRP-GFP, 916 treatment caused accumulation of both uncleaved (black arrow) and signal peptide-cleaved (red arrow) protein, which were the same size as bands observed for PEXEL R>A mutant KAHRP-GFP (Figure 3C, right). These bands were shown previously to be uncleaved and signal peptide-cleaved KAHRP-GFP, respectively, by mass spectrometry [11].

As 916 treatment caused accumulation of both uncleaved and signal peptide-cleaved species of PEXEL proteins, the potential for off-target effects against signal peptidase was investigated using parasites expressing SERA5s-GFP. This protein contains a signal peptide but lacks a PEXEL and is efficiently secreted to the parasitophorous vacuole (Figure S3B). 916 treatment did not impair processing of the signal peptide from SERA5s-GFP (Figure 3D, see position of black arrow), indicating it cannot inhibit signal peptidase. Taken together, this shows that 916 can effectively inhibit PMV, but not signal peptidase, in *P. falciparum*–infected erythrocytes and that 024 and 025 have no affect on PMV or signal peptidase activity at concentrations up to 50 µM.

### PMV Acts Cotranslationaly at the ER in *P. falciparum*

The rate of PEXEL protein synthesis, ER import, and processing by PMV in *P. falciparum* is unknown. We evaluated these processes by radiolabeling parasite proteins in culture for 0.5–15 min before immunoprecipitating PfEMP3-GFP with anti-GFP agarose, visualizing bands by autoradiography and quantifying them by densitometry. Labeled PfEMP3-GFP became evident 1 min after addition of label to the culture medium and increased exponentially throughout the experiment (Figures 3E and S3D). Uncleaved PfEMP3-GFP (black arrow) was faint and the major species was a doublet of approximately 33 kDa (red arrow; signal peptide-cleaved) and 29 kDa (blue arrow; PEXEL-cleaved; Figure 3E). This showed that signal peptidase cleaves PfEMP3 within seconds (<1 min) of protein synthesis (i.e., cotranslationaly), but this molecular species may be transient, as it was not detected by immunoblot of 916-treated parasites, or in PEXEL R>A mutant protein (Figure 3A–C). The radiolabeled bands on the ^35^S-membrane were confirmed to be GFP-specific by immunoblot (Figure S3C). PEXEL-cleaved PfEMP3-GFP (blue arrow) was also evident 1 min after addition of label to the culture medium and increased exponentially, indicating PMV cleavage was also rapid and likely cotranslational (Figures 3E and S3D). The proportion of PEXEL-cleaved protein increased slightly as signal peptide-cleaved protein decreased (Figure 3E), suggesting signal peptidase cleaves before PMV and that PMV may cleave after signal peptidase.

Addition of 916 to parasites for 5 h prior to radiolabeling caused accumulation of uncleaved PfEMP3-GFP in parasites (black arrow), which was evident 1–2 min postlabeling (Figure 3F). After a total of 15 min, the degree of PMV inhibition in parasites was quantified by densitometry, and a 13-fold decrease in PEXEL cleavage was observed compared to labeling without 916 (Figure 3G), indicating PMV was inhibited. The signal peptide-cleaved and PEXEL-cleaved species were only weakly detectable throughout the experiment and were not visible until 3–5 min postlabeling (Figure 3F), compared to a stronger signal within 1–2 min of labeling in the absence of inhibitor when the same quantity of parasites was used (Figure 3E). This indicated that 916 significantly blocked PMV in *P. falciparum* and caused a delay in protein synthesis, ER import, or N-terminal processing of PfEMP3-GFP.

Processing of uncleaved PfEMP3-GFP within the PEXEL was rescued after 15 min of culture in inhibitor-free medium (Figure S3E,F), indicating that PMV is able to process full-length PfEMP3-GFP.

### WEHI-916 Kills *P. falciparum* at the Ring-Trophozoite Transition

**Figure 4:**
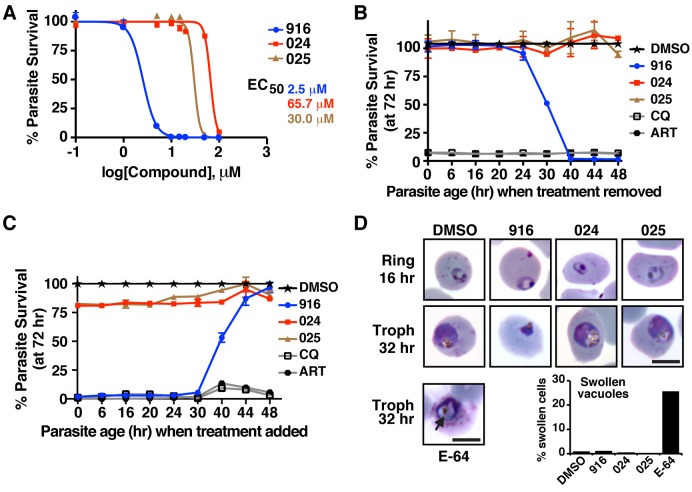
WEHI-916 is lethal to *P. falciparum* 3D7. (A) Dose-response curves of *P. falciparum* 3D7 in the presence of 916, 024, or 025. EC_50_ values are shown. (B) Parasitemia measured at 72 h (*y*-axis) following drug treatment at rings (30 min postinvasion) and replacement of the medium with inhibitor-free medium (wash-out) at the time intervals shown (*x*-axis). (C) Parasitemia at 72 h (*y*-axis) after replacement of inhibitor-free medium with media containing compounds at the intervals shown (*x*-axis). Parasitemia was determined by FACS in (A–C) and is relative to DMSO treatment in (B) and (C). Concentrations are as follows: 916, 024, 025 (15 µM); CQ, chloroquine (150 ng/ml); ART, artemisinin (100 ng/ml). Error bars in (A–C) are mean ±SEM from duplicate experiments. (D) Light micrographs of Giemsa-stained parasites 16 and 32 h after drug treatment at early rings (15 µM). 916-treated parasites failed to develop into trophozoites and did not recover. Ring parasites treated with E-64 (10 µM) [22] contained swollen food vacuoles (arrow) due to inhibition of proteases involved in hemoglobin degradation; however, treatment with DMSO, 916, 024, or 025 did not cause swelling. Swelling was quantified using 500 infected cells per condition in duplicate. Scale bar is 6 µm.

Having demonstrated that 916 can directly engage PMV in *P. falciparum*–infected erythrocytes, the effect of the inhibitor on parasite viability was examined by treating early ring parasites for 72 h and assessing parasitemia by flow cytometry. 916 killed parasites with half maximal effective concentration (EC_50_) of 2.5–5 µM (Figure 4A). Analogs 024 and 025 had negligible effect on parasite viability at concentrations up to 20 µM, where 916 completely killed parasites; however, they had EC_50_ values of 66 µM and 30 µM, respectively, indicating they adversely affected parasite growth at high concentrations. Because PEXEL processing in parasites was unaffected by 024 and 025 treatment, even at 50 µM (Figure 3C), we conclude that the analogs impart toxicity at high concentrations independent of PMV.

To determine the stage of the parasite lifecycle that 916 exerted its toxic effects, ring parasites were treated with 15 µM 916 for increasing times through the 48 h cycle and then cultured in inhibitor-free medium to a total of 72 h to see if parasites could recover. Parasites grown in 916 for 1–20 h completely recovered and grew like DMSO-treated controls; however, treatment for >23 h adversely affected growth (Figure 4B), indicating the timing of killing began after 20 h of age, at the ring-trophozoite transition. Analogs 024 and 025 did not affect growth at any parasite stage at the concentration used (15 µM), whereas chloroquine and artemisinin killed parasites when added to rings for only 30 min before addition of inhibitor-free medium (Figure 4B, see 0 h). This indicated the controls either killed rapidly (artemisinin) or were retained inside parasites and killed later, as chloroquine is reported to kill trophozoites [22],[23]. Both profiles were clearly different than that observed for 916.

Toxicity by 916 after the ring-trophozoite transition was then investigated by adding compound to parasites at different time points through the 48 h cycle. At 48 h, inhibitor-free medium was added and parasitemia for all conditions was determined at 72 h. Toxicity decreased when 916 was added to parasites aged beyond 24 h and schizonts were resistant, indicating 916 did not affect merozoite egress or reinvasion (Figure 4C). As expected, the addition of 916 to rings or early trophozoites was lethal (Figure 4C). 024 and 025 did not have any effect on parasite growth at the concentration used, whereas all parasite stages were sensitive to chloroquine and artemisinin (Figure 4C).

Light microscopy of parasites following treatment of early ring stages with 916 revealed a normal ring-stage morphology after 16 h; however, treatment for 32 h revealed a blockage in the ring-trophozoite transition and the majority of parasites appeared pyknotic and abnormal (Figure 4D). As greater than 50% of parasites could not recover from this treatment condition (refer to Figure 4B), the majority of parasites with this appearance were dying or dead. Treatment with DMSO, 024, or 025 had no effect on development by 32 h at 15 µM (Figure 4D). The morphology of parasites treated with 916, 024, 025, and DMSO was distinctly different from that observed for E-64–treated parasites, which contained swollen food vacuoles from inhibition of haemoglobin breakdown [24] (Figure 4D, arrow). This indicated that parasite toxicity to 916 was unlikely due to off-target inhibition of those food vacuole proteases. Collectively, the toxicity profile seen in the above experiments defines the window of parasite death as between 20 and 30 h, consistent with perturbed protein export and erythrocyte remodeling [7].

To gain further insight into the effects of 916, 024, and 025 on parasites, we assessed global protein synthesis following drug treatment by radiolabeling parasite proteins. Treatment of trophozoites with inhibitor for 5 h prior to radiolabeling had no detectable effect on translation, even at 50 µM concentrations (Figure S4A), indicating the compounds are not direct inhibitors of the translation machinery. We next assessed protein synthesis following 23 h of drug treatment of ring stage parasites (aged 1–3 h old at the initiation of treatment). A minor reduction in translation was observed following 916 treatment, but not 024 or 025 treatment, even at 50 µM (Figure S4B). A small but concomitant decrease in the cytosolic protein, Aldolase, was also evident by immunoblot following 916 treatment but not 024 or 025 treatment (Figure S4B), suggesting that parasites were beginning to die from the 916 treatment (parasites ranged from 24–26 h old at this time point). Indeed, Giemsa smears following treatment identified a small proportion of pyknotic parasites in the population (results not shown). Treatment with Brefeldin A, which prevents retrograde trafficking and ER exit, for 23 h severely impaired translation and parasites appeared as dying rings (i.e., had not progressed to trophozoites). It is possible that PMV inhibition by 916 treatment has a similar but weaker effect to BFA, in that it causes the accumulation of uncleaved PEXEL precursors in the ER, perturbing ER transport, and that this negatively affects translation by an ER stress response [25]. An alternative possibility is that translation was decreased slightly as a result of parasites dying from 916-mediated impairment of erythrocyte remodeling. Either way, the profile for 916 was different to that seen for 024 and 025, even at 50 µM, indicating the latter analogs likely kill parasites via a different mechanism to 916.

### Knockdown or Overexpression of PMV Modulates Parasite Sensitivity to WEHI-916

**Figure 5:**
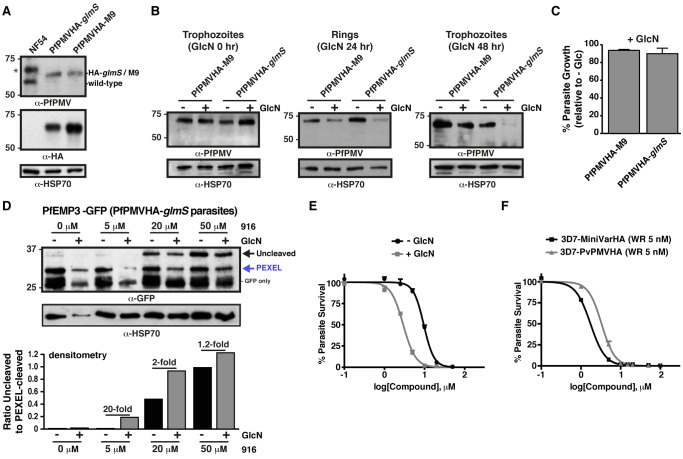
PMV knockdown or overexpression modulates sensitivity to WEHI-916. (A) Immunoblot with Rα-PfPMV antibodies shows successful integration of the PMVHA-*glmS* or -M9 plasmid (M9 is an inactive *glmS* riboswitch control). The upper band (α-PfPMV blot) in lane 1 (denoted by *) is nonspecific. The same blot is shown below, probed with α-HA antibodies. HSP70 is a loading control. (B) Knockdown of PMV in *P. falciparum* NF54 following 5 mM GlcN treatment. (Left) 0 h GlcN treatment of trophozoites causes no knockdown. (Center) The 24 h GlcN treatment of trophozoites causes ∼80% knockdown of PMV in subsequent rings compared to “−GlcN.” (Right) 48 h GlcN treatment of trophozoites causes ∼90% knockdown of PMV in subsequent trophozoites compared to “−GlcN.” A small degree of knockdown is seen for M9, indicating GlcN has a minor effect. (C) PMV knockdown by GlcN has no significant effect on parasite growth rate (*p* = .6250). Trophozoites were treated with 0 mM or 5 mM GlcN and parasitaemia determined 48 h later by flow cytometry. Data are % growth “+GlcN” relative to “−GlcN,” and data are mean ±SEM of a representative of duplicate experiments. (D) PEXEL processing of PfEMP3-GFP in *P. falciparum* parasites expressing PMVHA-*glmS* is reduced more by 916 treatment when PMV is knocked down [+GlcN (5 mM for 48 h prior to addition of 916)]. Densitometry shows the ratio of uncleaved to PEXEL-cleaved protein in each lane beneath the blot. Note that PfEMP3-GFP expression is lower in “+GlcN” parasites despite relatively similar HSP70 levels. (E) Dose-response curves of *P. falciparum* expressing PMVHA-*glmS* shows parasites have enhanced sensitivity to 916 following PMV knockdown (3.3-fold decrease in EC_50_). Parasitemia was determined 72 h after addition of 916 to ring parasites with or without PMV knockdown (knockdown ring parasites were obtained by adding 6 mM GlcN to trophozoites for 24 h). GlcN and 916 were maintained in the culture medium throughout. (F) Dose-response curves of *P. falciparum* overexpressing PvPMVHA or a mini PfEMP1HA reporter (miniVarHA) in the presence of 5 nM WR99210 show parasites have increased resistance to 916 when PMV is overexpressed (1.9-fold increase in EC_50_).

Treatment of *P. falciparum* with 916 impaired PEXEL cleavage and killed parasites, strongly suggesting that PMV is essential. To investigate this phenotype further, conditional protein knockdown was attempted in *P. falciparum* using the RNA-degrading *glmS* ribozyme, which utilizes glucosamine (GlcN) as a cofactor [26]. DNA encoding 3× HA epitopes, a stop codon, and *glmS* was incorporated in frame at the 3′ of the *PMV* locus by homologous recombination (Figure S5A). Correct genomic integration was confirmed by immunoblot with anti-HA and anti-PfPMV antibodies (Figure 5A). To activate *glmS*, GlcN was titrated into the culture medium of trophozoites. From 75% to 90% PMV knockdown was achieved after 48 h using 4–6 mM GlcN, but higher concentrations adversely affected parasite HSP70 levels and were subsequently avoided (Figure S5B). Addition of 5 mM GlcN to trophozoites for 24 h reduced PMV levels in subsequent rings by approximately 80% and trophozoites by ∼90% but caused little knockdown in parasites expressing inactive *glmS* (M9) (Figure 5B) [26]. Protein export predominates in rings, when knockdown reached ∼80%; surprisingly, this substantial degree of knockdown did not significantly affect PEXEL processing or parasite growth rate (*p* = .6250; Figure 5C), indicating that the remaining PMV levels were sufficient to enable export and sustain parasite development. This demonstrates that PMV activity is potent in *P. falciparum* and that knockdown to approximately 20% of wild-type levels could not facilitate the characterization of PMV essentiality.

As 916 inhibited PMV in parasites (for example, by 13-fold, Figure 3G), the additive effect of PMV knockdown plus 916 treatment was investigated. Parasites expressing PMVHA-*glmS* were transfected with a construct encoding PfEMP3-GFP, and PEXEL processing was assessed by immunoblot. PEXEL processing of PfEMP3-GFP was barely affected by 48 h of PMV knockdown alone (Figure 5D, see “+GlcN,” 0 µM 916); however, addition of 916 to parasites for 5 h impaired PEXEL cleavage and this was significantly enhanced following knockdown of PMV (e.g., by 50% at 20 µM; Figure 5D). The quantity of PfEMP3-GFP expressed in the PMV knockdown (+GlcN) appeared slightly less than in parasites without knockdown (−GlcN), whereas the loading control HSP70 did not vary appreciably between conditions (Figure 5D).

Parasites expressing PMVHA-*glmS* were next assessed for toxicity to 916. The EC_50_ of 916 was reduced by 3.3-fold following PMV knockdown compared to no knockdown (Figure 5E). As a control, parasites expressing PMVHA-M9 were treated with 916 in the presence or absence of GlcN; the EC_50_ reduced by 1.4-fold in the presence of GlcN, indicating it had a minor effect. However, the enhancement of PEXEL cleavage inhibition and 3.3-fold sensitization of parasites to inhibitor following knockdown indicated that PMV is a direct target of 916 and that PMV inhibition is lethal to parasites.

We next investigated the possible effects of PMV overexpression on parasite sensitivity to 916. Although parasites expressing PfPMVHA do not overexpress enzyme, due to integration of the construct at the endogenous *PMV* locus [13], parasites expressing PvPMVHA from episomes also express wild-type levels of endogenous enzyme (Figure 1B,C) and therefore contain additional, active PMV in the ER. To control for the carriage of episomes and selection on WR99210, sensitivity to 916 was compared between parasites overexpressing a similar construct on episomes on WR99210 selection (encoding miniVarHA; see Figure 1F). The EC_50_ of 916 was 1.9-fold greater for parasites overexpressing PvPMVHA compared to parasites overexpressing the control construct, and 1.4-fold greater than wild-type 3D7 parasites without WR99210 selection, indicating that PMV overexpression had increased parasite resistance to 916 (Figure 5F).

### PMV Inhibition Impairs Protein Export, PfEMP1 Display, and Cytoadherence

**Figure 6:**
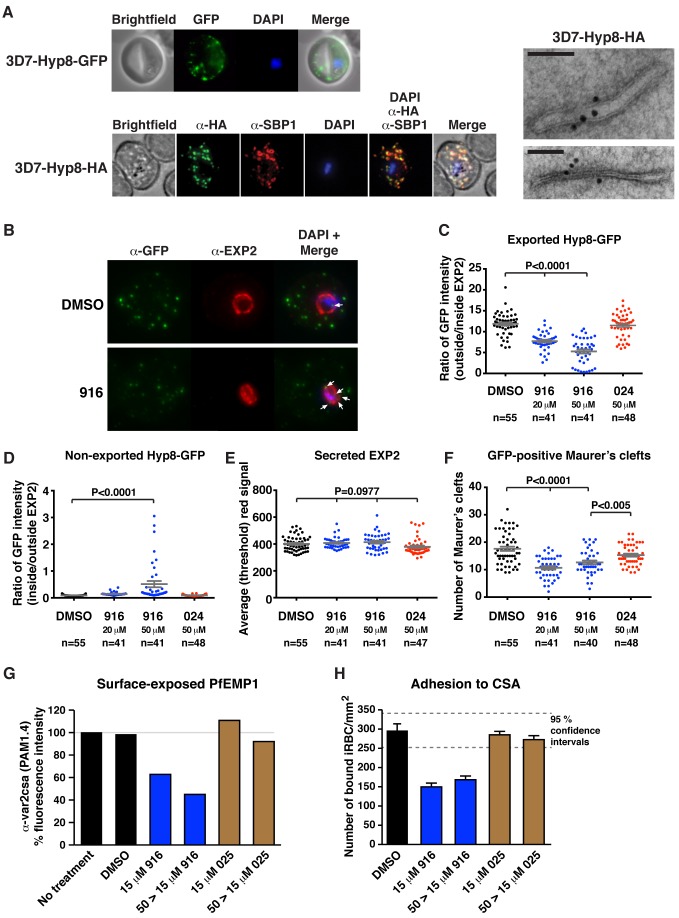
PMV inhibition impairs protein export, PfEMP1 display, and cytoadherence. (A) (Top) Immunofluorescent micrographs show Hyp8-GFP is exported and localizes within puncta in the infected erythrocyte. (Middle) Hyp8-HA localizes within SBP1-containing MCs. (Right) Immunoelectron microscopy shows Hyp8-HA localization at MCs. Scale bar is 100 nm. (B) Maximum intensity projection micrographs showing export of Hyp8-GFP to MCs and secretion of EXP2 to the parasitophorous vacuole membrane following treatment with DMSO or 916 (50 µM). Puncta of nonexported GFP within the parasite and vacuole is shown (arrows). (C) GFP intensity in MCs (outside EXP2 signal) and within the parasite and parasitophorous vacuole (inside EXP2 signal) was quantified following drug treatment (see Materials and Methods) and is presented as a ratio: exported = ratio outside/inside EXP2. The number of infected cells counted (*n*) is shown. (D) GFP within the parasite and parasitophorous vacuole (inside EXP2 signal) was quantified across treatments and is presented as a ratio: nonexported = ratio inside/outside EXP2. (E) EXP2 intensity in the parasitophorous vacuole membrane (red) was quantified across treatments and is presented as average (threshold) signal. (F) The number of GFP-positive MCs per infected erythrocyte was quantified across treatments. Error bars represent the mean ±SEM, and *p* values were determined by ANOVA in (C–F). (G) Surface-exposed PfEMP1 (VAR2CSA) on infected erythrocytes was measured following inhibitor treatment by FACS using monoclonal human PAM1.4 serum [30]. Geometric mean fluorescence of >100,000 cells per condition are shown relative to no treatment. Parasites received inhibitor at 15 µM for 23 h (15 µM) or 50 µM for 12 h followed by a reduction to 15 µM for 11 h (50>15 µM). Shown is a single representative of duplicate experiments. Raw FACS data are presented in Figure S7. (H) Adhesion of infected red blood cells (iRBCs) to chondroitin sulfate A (CSA) under static conditions. Adherent iRBCs were counted in ten 0.28 mm^2^ fields of view per sample from triplicate samples and are shown as number of iRBCs per mm^2^. Shown is a single representative of duplicate experiments. Data represent mean ±SEM. 95% confidence intervals are shown (grey dashed lines). 916 significantly reduced adhesion to CSA *p*<.0001.

Localization of parasite proteins in Maurer's clefts (MCs), which are parasite-induced membranous structures in the erythrocyte that facilitate protein trafficking, enables accurate quantification of export by immunofluorescence microscopy as the signal is concentrated in puncta [27]. To study export in *P. falciparum*, we investigated a novel PEXEL-containing protein with two transmembrane domains and unknown function, called Hyp8 (MAL13P1.61/PF3D7_1301700) [14],[28], that we hypothesized may localize to MCs. Transgenic parasites expressing Hyp8-GFP or Hyp8-HA were generated (Figure S6A). Immunoblotting revealed that Hyp8 is expressed in rings (Figure S6B), and immunofluorescence microscopy showed it is exported (Figure S6C) and colocalizes with SBP1 in MCs (Figure 6A). Immunoelectron microscopy confirmed that Hyp8 localizes in MCs (Figure 6A, right). Three independent attempts to delete the *hyp8* gene were unsuccessful in this study, in addition to earlier reported attempts [17], suggesting that Hyp8 may be an essential exported protein.

The effect of 916 treatment on export in *P. falciparum*–infected erythrocytes was then examined. Early ring parasites were treated with inhibitor, and the subcellular Hyp8-GFP fluorescence was quantified by immunofluorescence microscopy (Figure 6B). 916 treatment caused a dose-dependent decrease of Hyp8-GFP in MCs (the GFP signal in puncta outside the EXP2-labelled parasitophorous vacuole membrane) compared to DMSO and 024 treatment (*p*<.0001; Figure 6C). A small but significant increase in nonexported GFP signal (the signal inside the EXP2 labeling) was observed as puncta of fluorescence internal to the parasitophorous vacuole membrane following 916 treatment (*p*<.0001; Figure 6D, see also arrows in B).

We next examined whether 916 treatment affected protein secretion in *P. falciparum*–infected erythrocytes by measuring the quantity of EXP2 signal at the parasitophorous vacuole membrane following treatment. There was no statistical difference in EXP2 signal between treatments (*p* = .0977; Figure 6E), indicating 916 specifically affected export but not secretion, under the conditions used.

As Hyp8 localizes to MCs, we quantified the number of GFP-positive MCs in infected cells following the drug treatments. The mean number of clefts was significantly reduced by 916 treatment, by up to 39%, compared to DMSO (*p*<.0001; Figure 6F). Collectively, these data demonstrated that 916 dramatically reduced the export of the PEXEL protein, Hyp8, and that MC development was impaired following treatment.

An important function of exported proteins in remodeling and virulence is to assemble the cytoadherence complex at the erythrocyte surface [17]. Because export of Hyp8 and MC formation was decreased following PMV inhibition, we investigated whether trafficking of PfEMP1 was also affected by quantifying its display on the surface of infected erythrocytes. Ring-stage CS2-GFP parasites [29] selected for expression of the PfEMP1 *var2csa* gene were treated with one of two sublethal doses of 916 (Figure S7A), and surface-expressed PfEMP1 was measured 24 h postinvasion using PAM1.4 antibodies [30] that specifically recognize VAR2CSA [31] by flow cytometry. PfEMP1 surface expression decreased in a dose-dependent manner, by up to 55%, following 916 treatment, but addition of DMSO and 025 had no effect (Figure 6G). Parasitemia across all treatment conditions was measured as GFP fluorescence by flow cytometry and was approximately equal at 24 h, confirming parasite viability (Figure S7B).

To evaluate whether decreased PfEMP1 surface expression affected cytoadherence of infected erythrocytes, static binding assays with purified CSA were performed [32],[33]. Adhesion to CSA was reduced by almost 50% following treatment with 916 compared to DMSO (*p*<.0001) and 025 had no effect (Figure 6H). Collectively, this experimentally validates PMV activity as essential for export of PEXEL-containing proteins, resulting in correct MC formation and PfEMP1 assembly at the erythrocyte surface and cytoadherence.

## Discussion

Protein export allows malaria parasites to remodel their cellular niche, and the protein machineries involved are obvious targets for the development of inhibitors. PMV acts by cleaving the PEXEL in the parasite ER and represents one such target. We developed a PEXEL-mimetic compound that potently inhibits the activity of PMV and, combined with protein knockdown or overexpression, used it to demonstrate the essentiality of PMV for parasite survival and its function for export.

The PMV inhibitor 916 mimics the transition-state of amide bond proteolysis for PEXEL substrates using statine. Our PfPMV structural model in complex with 916 outlines key interactions that are likely necessary for inhibitor binding: the guanidine side chain of Arg forms salt bridges with the acid of Glu179 and 215, and a π-stacking interaction with Tyr177 in the S_3_ pocket of PMV. This in part explains the necessity for Arg at P_3_ for PEXEL processing. The P_1_ Leu side chain is encased by hydrophobic residues in the S_1_ pocket formed in part by Ile116, Tyr177, and Val227, explaining the importance of Leu in PMV binding.

916 potently blocked PMV activity *in vitro* and reduced PEXEL cleavage, by up to 13-fold (Figure 3G), in cultured parasites, demonstrating the inhibitor directly engaged PMV in the ER. However, inhibition was time-dependent and incomplete at even 50 µM, indicating the inhibitor has suboptimal qualities. This may be due to a combination of poor diffusion across membranes, suboptimal final concentration in the ER, and the potent activity of PMV in parasites, revealed in this study by knockdown of PMV protein levels. Although further work is required to develop an inhibitor with enhanced properties, 916 has proven sufficiently active in parasites to examine PMV function and essentiality.

Previously, it has been shown that overexpression of a PfPMV D118A mutant produced a dominant-negative effect on parasite growth rate and protein export [12]. When we overexpressed an alternate PfPMV mutant (D118A, D365A, F370A) on episomes in *P. falciparum*, we observed a similar defect in parasite growth rate and subsequent down-regulation of endogenous PfPMV expression levels, suggesting a negative feedback effect. A similar negative feedback effect has been described for *Toxoplasma gondii* myosin A [34]. Collectively, these PMV dominant-negative mutants provide evidence that the enzyme is important for parasite survival.

The effects of 916 were amplified when PMV was knocked down and decreased when PMV was overexpressed, demonstrating that PMV is a target and that its inhibition is toxic to parasites. Addition of 916 to ring stages arrested their transition to trophozoites, between 20 and 30 h postinvasion, and parasites could not recover. Although this phenotype is consistent with death from impaired export and cellular remodeling, it is possible that ER stress due to accumulation of uncleaved PEXEL proteins in the organelle, and a decrease in translation, contributed [25]. The morphology of parasites treated with 916 was distinctly different to E-64–treated parasites, which contained swollen food vacuoles from inhibition of haemoglobin breakdown [24]. This indicates that parasite toxicity to 916 was unlikely due to off-target effects on food vacuole proteases. Although aspartyl protease inhibitors are known to kill *P. falciparum*, it is not entirely clear which aspartyl proteases (Plasmepsins) are essential (reviewed in [35]). In *Plasmodium*, there are 10 Plasmepsins; I–IV are important enzymes for haemoglobin degradation by *P. falciparum*, but the genes encoding each enzyme can be deleted from the genome, indicating they are not essential [36]. The death of *P. falciparum* following 916 treatment is therefore unlikely to be due to inhibition of these Plasmepsins. A survey of *P. falciparum* transcriptomes [37] suggests that, of the remaining five Plasmepsins (VI–X), only VII, IX, and X are expressed by asexual blood-stage parasites; however, VII and X are expressed at very low levels. Thus, Plasmepsin IX (PMIX) is considered the primary possible off-target in our study. However, we have shown previously that HA-tagged PMIX does not cleave the PEXEL motif [13], and although the enzyme itself possesses a PEXEL motif, its function and essentiality is currently unknown.

Analogs of 916 that mimic noncleavable PEXEL mutant sequences (R>K, L>I) were ineffective inhibitors of PMV *in vitro* and had no discernable effect on parasites at concentrations that 916 inhibited PEXEL cleavage in parasites and was lethal (i.e., <20 µM). However, at concentrations above 20 µM they were toxic to parasites and possessed EC_50_ values between 6- and 26-fold less potent than 916. At 50 µM, we saw no evidence of PMV or signal peptidase inhibition, export or secretion defects, or global effects on translation caused by 024 or 025. This suggests that they hit a target(s) distinct from 916.

The rapid rate of protein synthesis, ER import, signal peptide processing, and PEXEL cleavage in *P. falciparum* was determined for the first time. The rate was within immeasurable seconds after translation, consistent with both signal peptidase and PMV activity occurring cotranslationaly. The full-length PEXEL is thus only present in the proprotein very transiently, making its function in export even more remarkable. This underscores the importance of the remaining PEXEL residues (xE/Q/D) in export following processing [11]. The PEXEL has been suggested to function independent of PMV by binding PI3P in the ER, and ER-derived transport vesicles, via the PEXEL Arg [38]. The rate at which PEXEL processing occurred in our experiments is inconsistent with this hypothesis, as the PEXEL Arg is cleaved off during, or soon after, ER entry. It is also challenging to envisage how the PEXEL Arg could dock within the S_3_ pocket of PMV, where it is required for proteolytic cleavage, if it is bound to the ER membrane via an interaction with PI3P.

This work has characterized a novel PEXEL protein, Hyp8, which is exported in the early ring-stage to MCs. The function of Hyp8 is unknown, but the *hyp8* gene was refractory to deletion and may be essential. 916 treatment dramatically impaired Hyp8 export, resulting in some accumulation in the parasite and possibly degradation. It is unknown whether the reduced export of Hyp8 directly contributed to parasite death; however, the phenotype demonstrates the importance of PMV in export of PEXEL-containing cargo. This is further supported by the decrease in MC numbers observed following 916 treatment; MC formation is known to require exported proteins (reviewed in [20]). The secretion of EXP2 to the parasitophorous vacuole membrane was unaltered by 916 treatment, demonstrating that the effects of 916 were specific to export. A clear defect in PfEMP1 surface exposure and cytoadherence was also observed following 916 treatment. PfEMP1 is unlikely to be a PMV substrate [18], but its trafficking through the erythrocyte and onto the surface requires at least six PEXEL-containing proteins [17]; thus, the demonstration that PMV activity is essential for PfEMP1 surface expression and cytoadherence is consistent with the current literature and validates the specificity of the inhibitor. Further, it directly demonstrates the importance of PMV at the first step in the export pathway for cellular remodeling that leads to virulence.

*P. vivax* is an important global pathogen that cannot be cultured in the laboratory, and novel therapeutic targets for this enigmatic parasite are urgently needed. This work has characterized PMV from *P. vivax* for the first time. PvPMVHA possesses the trafficking information to localize to the ER and has similar PEXEL cleavage activity and specificity to PfPMV. This indicates that PMV function is to cleave the PEXEL motif of exported proteins across *Plasmodium* spp., and future compounds that block PMV are likely to affect multiple *Plasmodium* spp. Protein export also occurs in gametocytes [28] and liver stages [39], and 916 may aid the characterization of PMV in these stages.

A putative PMV homolog, ASP5, is present in *Toxoplasma* and localizes to the Golgi [40]. Recent evidence suggests that some exported *T. gondii* proteins contain a PEXEL [41], and some are cleaved in a manner that requires the conserved PEXEL residues [42]. The PEXEL protease may therefore be conserved beyond the *Plasmodium* genus, and PMV and its homologs may therefore represent multistage, multispecies antiparasitic targets of the future.

## Materials and Methods

### Plasmids, Parasites, and Antibody Production

*P. falciparum* 3D7 parasites expressing PfPMVHA, PfPMVmutHA, and PfEMP3-GFP were generated previously [13], as was KAHRP-GFP [18] and CS2-GFP [29]. DNA encoding PvPMV or PvPMVmut fused to 3× HA tags was synthesized (Epoch Biosciences) and cloned into pGlux.1 [11] with *Xho*I and *Pac*I, removing GFP. DNA encoding miniVarHA [PfEMP1 NTS (residues 1–51) fused to SVL-TM-ATS (residues 2640–2734) of IT4 VAR2CSA] was synthesized (Epoch Biosciences) and cloned into pGlux.1 with *Xho*I and *Pac*I. DNA encoding the signal peptide of SERA 5 (PFB0340c) (residues 1–25) or the entire *hyp8* gene (MAL13P1.61) was amplified from *P. falciparum* gDNA and cloned in frame with GFP in pGlux.1 using *Xho*I and *Xma*I. For HA tagging Hyp8, the 3′ 800 bp of *hyp8* was cloned into p1.2-SHA [13] (also called pHA3; [43]) using *Bgl*II and *Pst*I. For tagging PfPMV with HA-*glmS* in *P. falciparum* NF54, the 3′ 1144 bp of *PMV* was cloned into pPTEX150-HA-*glmS*, which consisted of the *glmS* riboswitch from pGFP_*glmS* [26] cloned into pHA3 using *Bgl*II and *Pst*I to replace the *PTEX150* gene with *PMV*, generating pPMVHA-*glmS*. For tagging PfPMV with HA-M9, the M9 insert from pGFP_M9 [26] was cloned into pPMVHA-*glmS* to generate pPMVHA-M9. To express PfEMP3-GFP in *P. falciparum* NF54 harboring PMVHA-*glmS*, the dihydrofolate reductase selection cassette in pPfEMP3Glux.1 [13] was replaced with blasticidin deaminase using *BamH*I and *Hind*III prior to transfection. *P. falciparum* transfectants were selected with 5 nM WR99210 (Jacobus Pharmaceuticals) and/or 2 µg/ml Blasticidin S (Calbiochem) and grown in O+ human erythrocytes as described [18]. CS2-GFP parasites preferentially expressing the *var2csa* gene (PFL0030c/PF3D7_1200600) were selected every 2 wk by enriching for knob-positivity with gelatin [44] and panning for CSA-binding [45]. Rα-PfMV antibodies were generated by immunization of rabbits with recombinant PfPMV generated previously [13] and collecting serum during four boost immunizations. Affinity-purified polyclonal rabbit α-Hyp8 antibodies were generated by Genscript using the peptide N-^55^ETEQSTPAKPEPTE^68^-C.

### PMV-Agarose, PEXEL Cleavage Assays, Mass Spectrometry, and Parasite Growth Assays

PMV-agarose was prepared by adding α-HA-agarose (Sapphire Bioscience) to parasite lysates, prepared by sonication in 1% Triton X-100/PBS, for 1 h before extensive washing in same [12],[13]. PEXEL cleavage assays (20 µl total volume) consisted of 0.2 µl PMV-agarose in digest buffer (25 mM Tris, 25 mM MES, pH 6.4) with 1.5 µM FRET peptide substrate (DABCYL-RNKRTLAQKQ-E-EDANS, DABCYL-RNKATAAQKQ-E-EDANS, LifeTein; DABCYL-RNKKTLAQKQ-E-EDANS, DABCYL-RNKRTIAQKQ-E-EDANS; Mimotopes) ± inhibitor. Samples were excited at 340 nm and fluorescence emission measured at 492 nm using an Envision fluorescence plate reader (Perkin-Elmer) heated to 37°C for 150 min. Samples were shaken between measurements. For determination of the peptide cleavage position by PvPMVHA, the fluorogenic peptide (DABCYL-RNKRTLAQKQ-E-EDANS), representing the wild-type KAHRP PEXEL sequence, was incubated with and without PvPMVHA at 37°C for 48 h. Products of the incubation were detected by a molecular formula algorithm using an Agilent 6200 TOF/6500 series mass spectrometer.

Parasite growth assays were performed in 96-well plates by incubating highly synchronous ring-stage *P. falciparum* 3D7 or NF54 parasites with compounds solubilized in DMSO at the indicated concentrations for the indicated times. In the case of dose-response curves, medium was kept for the entire experiment; in the case of curves in Figure 4B,C, medium was replaced with inhibitor-free medium at 48 h postinfection. Parasitaemia was always determined at 72 h by flow cytometry. To knock down PMV, GlcN (Sigma) was added to trophozoites and drug curves initiated by adding compound at subsequent rings (24 h) for 24–72 h.

### Immunoblots

Trophozoites (30–34 h) expressing PfEMP3-GFP, KAHRP-GFP, or SERA5s-GFP were magnet-purified (Miltenyi Biotech), incubated with inhibitors in 400 µl total volume at 37°C for 1–5 h, treated with 0.09% saponin containing inhibitor, and washed pellets were solubilized in Laemmli's buffer, boiled for 3 min, and frozen at −20°C. Proteins were separated by SDS-PAGE, transferred to nitrocellulose and blocked in 10% skim milk/PBS-T, and probed with rat α-HA (Roche 3F10; 1∶1,000), mouse α-GFP (Roche; 1∶1,000), rabbit α-Aldolase (1∶1,000), rabbit α-HSP70 (1∶4,000), rabbit α-PfPMV (1∶1,000), or rabbit α-Hyp8 (1∶500) primary antibodies followed by horseradish peroxidase-conjugated secondary antibodies (Silenius) and detected by enhanced chemiluminescence (Amersham).

### Protein Radiolabeling, Pulse-Chase, and Densitometry

Whole parasite proteins were radiolabeled by culturing magnet-purified trophozoites (wild-type 3D7 or expressing PfEMP3-GFP) in Met/Cys-free medium for 30 min at 37°C before addition of 800 µCi/ml ^35^S-Met/Cys (Perkin/Elmer) to the medium for the indicated times. Pellets were snap frozen in ethanol/dry ice bath and stored at −80°C. For radiolabeling in the presence of PMV inhibitor, parasites were treated with 20 µM WEHI-916 for 5 h before labeling commenced. For pulse-chases, proteins were radiolabeled in the presence or absence of inhibitor, as above, before further culture in radiolabel-free, inhibitor-free complete medium for the indicated times at 37°C before snap freezing. Frozen samples were either solubilized in Laemmli's buffer (Figure S4) or solubilized in 1% Triton X-100/PBS with protease inhibitor cocktail (Roche) and PfEMP3-GFP species immunopurified with α-GFP agarose (MBL) at 4°C for 2 h (Figures 3 and S3), and proteins were resolved by SDS-PAGE, visualized by autoradiography (7-d exposures), and quantified using a GS-800 Calibrated Densitometer (Bio-Rad).

### Microscopy, Quantification of Hyp8 Export, and Statistical Analyses

For immunofluorescence microscopy, smears were fixed in cold acetone∶methanol (90∶10) and probed with rabbit Rα-PfPMV (1∶750), mouse Mα-PfPMV (1∶25), rabbit α-EXP2 (1∶200), rat α-HA (Roche 3F10; 1∶50), mouse α-GFP (Roche; 1∶500), or rabbit α-Hyp8 (1∶200) antibodies followed by Alexa Fluor 488- or 594-conjugated secondary antibodies (Molecular Probes; 1∶1,000). DNA was stained with 4′-6-Diamidino-2-phenylindole (DAPI) at 0.2 µg/ml. Samples were viewed on a Deltavision Elite microscope and images collected with a Coolsnap HQ2 CCD camera through an Olympus 100× UPlanSApo NA1.4 objective with SoftWorx software. Images were assembled with ImageJ Fiji 1.47d and Adobe Photoshop CS6 v13.0 x64. Light and immunoelectron microscopy was performed as described in [46].

For quantification of events in cells infected with parasites expressing Hyp8-GFP, highly synchronous ring-stage parasites engineered to express Hyp8-GFP from the *CRT* promoter were obtained by incubation of erythrocytes with viable merozoites for 15 min [47] and treated with 20 or 50 µM 916 30 min postinvasion for 13 h (until Hyp8-GFP expression from the *CRT* promoter had occurred for 1 h). Smears were fixed in 90∶10 acetone∶methanol, labeled with anti-GFP and anti-EXP2 antibodies, and Z-stacks captured on a Deltavision Elite microscope using a 100× objective. Over 40 Z-stacks per condition were imaged using the same exposure settings to allow quantitative analysis between groups.

#### Hyp8 export

A parasite mask was generated by combining the signals from the maximum intensity projections of the nuclear (DAPI) and parasitophorous vacuole membrane (EXP2) images and setting a manual threshold. A whole infected erythrocyte mask was produced by manually tracing the cell boundary using the DIC image. Average GFP intensity measurements were determined on masked summed intensity projections of the GFP images using MetaMorph Image Analysis suite (Molecular Devices, USA). Ratios of the average intensity measurements of GFP inside the parasite (inside EXP2) versus exported to the erythrocyte (outside EXP2) were determined.

#### EXP2 secretion

The EXP2 signal was measured by masking the infected cell using a watershed transform on the summed and filtered red signal, seeded by the DAPI-positive parasite.

#### Number of MCs

Clefts were counted using ImageJ's “Find Maxima” function to detect local maxima in the green signal within the masked cell region.

#### Statistics

Kinetics and statistical analyses were performed with GraphPad Prism 6.0b. Single comparisons were performed using *t* test and multiple comparisons by ANOVA.

### 916 Dosage Regime and PfEMP1 Surface Display

As 916 treatment has adverse affects on parasites after 24 h, a sublethal dosing regime was developed (see Figure S7) to maximize the inhibitor effect while ensuring parasites remained viable 24 h postinvasion when surface-exposed PfEMP1 was measured. Treatment with >15 µM for 23 h or with 50 µM for >12 h postinvasion prior to decreasing to 15 µM adversely affected parasite growth and was avoided.

To measure PfEMP1 display, highly synchronous ring-stage CS2-GFP parasites preferentially expressing VAR2CSA (see Plasmids, Parasites, and Antibody Production) were obtained by incubation of erythrocytes with viable merozoites for 15 min [47], and parasites were treated with 916, 025, or DMSO using the dosage regime above. At 24 h postinvasion (presence of inhibitor for no more than 23 h), erythrocytes were incubated with human monoclonal PAM1.4 serum [30] (1∶200) to label VAR2CSA followed by goat anti-human IgG Biotin-conjugated secondary antibodies (Invitrogen) (1∶200) and Alexa Fluor-633 Streptavidin-conjugated tertiary antibodies (Invitrogen) (1∶500) for 30 min each. Labeled cells were washed with 0.1% casein/PBS and analyzed on a FACSCalibur cytometer (Becton-Dickinson, USA). Fluorescence in channel FL1 was used to measure parasite-infected erythrocytes (GFP), and fluorescence in channel FL4 was used to measure bound PAM1.4 IgG antibodies (Alexa 633) for each sample. The geometric mean fluorescence of uninfected erythrocytes (treated with secondary and tertiary but not primary antibodies) was deducted from the geometric mean fluorescence of infected erythrocytes using >100,000 cells per condition. Experiments were conducted in duplicate. Analyses were performed using FlowJo 8.8.7 (Tree Star, USA).

### CSA Adherence Assay

Adhesion assays were performed as described previously [32],[33]. Briefly, CSA (Sigma) was spotted at 50 µg/ml in triplicate into petri dishes, incubated overnight at 4°C, and blocked in 1% casein/PBS for 2 h. Inhibitor-treated erythrocytes were added to CSA-coated dishes and incubated for 45 min at 37°C. Dishes were washed four times with 5 ml warm RPMI-HEPES, fixed in 2% paraformaldehyde for 2 h, stained with 10% Giemsa for 15 min, and the number of adherent erythrocytes per mm^2^ quantified by light microscopy counts. Assays were performed in triplicate.

### PMV Modeling and Inhibitor Synthesis

This information is presented in Materials and Methods S1.

## Supporting Information

Figure S1 ClustalW alignment of PMV from *P. falciparum* and *P. vivax*. The putative *P. vivax* PMV protein sequence (PvPMV; PVX_116695) was identified by homology searches using the *P. falciparum* PMV sequence (PfPMV; Pf3D7_1323500). A ClustalW alignment shows they share 82.2% similarity (485/590) and 54.7% identity (323/590) over the full-length sequences including gaps. The predicted signal peptide is shown below the red line, the catalytic dyads are shown in bold with catalytic aspartic acid residues below the red circles, and the C-terminal transmembrane domain is shown below the black line. Four insertions in PfPMV are absent from PvPMV, accounting for their ∼7.5 kDa size difference.
(TIF)

Figure S2 Purity, substrate cleavage position, and kinetics of PMVHA-agarose. (A) Immunoblot of immunopurified PfPMVHA or PvPMVHA eluted from α-HA-agarose with reducing sample buffer shows that the sample preparations contain PMVHA that are species-specific. (Left) α-HA antibodies show purification of PfPMVHA (69.1 kDa once signal peptide removed) and PvPMVHA (60.9 kDa once signal peptide removed) from parasite lysates. (Right) The blot on the left was stripped and reprobed with Rα-PfPMV antibodies. Only HA-tagged PfPMV but not endogenous wild-type PfPMV (64.4 kDa once signal peptide removed) was present in the PfPMV-agarose preparation (lane 1). In lane 2, the Rα-PfPMV antibody does not cross-react with PvPMVHA (60.9 kDa) and does not identify any endogenous wild-type PfPMV in the PvPMVHA-agarose preparation. The black spot on the right of the blot (*) is a nonspecific artefact, not PvPMVHA. (B) LC chromatogram (214 nm) of the fluorogenic KAHRP PEXEL peptide after incubation at 37°C for 48 h with PvPMVHA (red trace) and without PvPMVHA (blue trace). The products of processing by PvPMVHA can be observed: the C-terminal cleavage product NH_2_-AQKQ-E(EDANS)NH_2_ (R*f* 0.66 min) and the N-terminal fragment DABCYL-RNKRTL-OH (R*f* 1.7 min) are labeled. The unprocessed fluorogenic peptide DABCYL-RNKRTLAQKQ-E(EDANS)NH_2_ is shown to possess an approximately similar R*f* (1.7 min) to the processed N-terminal fragment. MS-TOF analysis shows (C) the C-terminal cleavage product (↓AQKQ-E-EDANS), (D) the N-terminal cleavage product (DABCYL-RNKRTL↓), and (E) the unprocessed fluorogenic KAHRP peptide. (F) Michaelis–Menten curve showing the rate of cleavage (relative fluorescence units per min) of increasing concentrations of fluorogenic KAHRP PEXEL peptide by PfPMVHA (squares) and PvPMVHA (circles) after 2 h. The data were used to derive *Km* values reported in the text.
(TIF)

Figure S3 WEHI-916 inhibits PMV in *P. falciparum*. (A) Immunoblot with anti-GFP antibodies shows that cleavage inhibition of PfEMP3-GFP by 916 treatment (20 µM 916 for 4 h) is rescued after culture in inhibitor-free medium for the indicated times. Densitometry of the uncleaved band is shown below each lane, and HSP70 is a loading control. (B) Immunofluorescence micrograph of *P. falciparum* expressing SERA5s-GFP, which lacks a PEXEL, showing the chimera is secreted to the parasitophorous vacuole. (C) Immunoblot of the ^35^S-membrane in Figure 3E with anti-GFP antibodies confirms the uncleaved (black arrow), signal peptide-cleaved (red arrow), PEXEL-cleaved (blue arrow), and GFP-only bands in Figure 3E are indeed GFP-specific. (D) Modified exposure of the blot in Figure 3E, showing the presence of signal peptide-cleaved (red arrow) and PEXEL-cleaved (blue arrow) protein 1 min after addition of label to the culture medium. (E) Pulse chase of PfEMP3-GFP in *P. falciparum*–infected erythrocytes. (Left) Radiolabeling of PfEMP3-GFP for 5 min (pulse) followed by culture in label-free, inhibitor-free medium for the indicated times (chase) revealed little uncleaved protein (black arrow) and that signal peptide-cleaved protein was more abundant than PEXEL-cleaved protein. The proportion of PEXEL-cleaved protein increased after 3 min of the chase, as the proportion of signal peptide-cleaved protein decreased, demonstrating that PMV can cleave the signal peptide-cleaved protein. (F) Accumulation of uncleaved radiolabeled PfEMP3-GFP in parasites following 5 h of 916 pretreament (20 µM) was reduced after 5–15 min of the chase in label-free, inhibitor-free medium. A concomitant increase in the proportion of PEXEL-cleaved protein was observed from 5 min onward as the quantity of uncleaved protein sharply decreased, demonstrating that PMV can cleave the full-length protein.
(TIF)

Figure S4 Effect of WEHI-916 on global protein translation in *P. falciparum*. (A) Magnet-purified trophozoites treated with DMSO or 916, 024, 025 (15 or 50 µM), or Brefeldin A (BFA; 10 µg/ml) for 5 h prior to labeling parasite proteins with ^35^S-Methionine/Cysteine for 15 min reveal no defect in global protein synthesis. Aldolase levels were examined by immunoblot of the same blot as a loading and viability control. (B) Ring parasites treated with DMSO or 916, 024, 025 (15 µM or 50 µM for 12 h followed by 15 µM for 11 h (50>15 µM)) or BFA (5 µg/ml) for 23 h prior to labeling parasite proteins with ^35^S-Methionine/Cysteine for 15 min reveals no defect in global protein synthesis for DMSO, 024, or 025. A slight reduction in translation is seen with 916 treatment, and immunoblotting revealed that Aldolase levels were also reduced following 916 treatment, suggesting either that accumulation of uncleaved PEXEL precursors in the ER has a negative effect on translation or that a proportion of parasites were beginning to die or both. BFA treatment, which halts retrograde transport and ER exit, severely reduced translation in parasites and Aldolase levels, suggesting negative feedback on protein synthesis occurs in parasites with blocked ER transport. The effect of 916 was clearly different to that of 024 and 025, suggesting the latter impart toxicity independent of PMV at concentrations >20 µM.
(TIF)

Figure S5 Effect of GlcN addition to *P. falciparum* cultures. (A) Schematic of allelic exchange to introduce 3× HA epitopes and *glmS* riboswitch into the 3′ of the *PMV* gene in *P. falciparum* NF54. (B) *P. falciparum* trophozoites were treated with 0–10 mM GlcN for 48 h and PMV levels assessed by immunoblot with α-PfPMV antibodies. HSP70 was used as a loading control and to assess parasite viability in the presence of GlcN. Densitometry of the PMV and HSP70 bands is shown below the blots. We utilized 4–6 mM GlcN for all future experiments.
(TIF)

Figure S6 Hyp8 epitope tagging, expression, and export. (A) Schematic of Hyp8-GFP and Hyp8-HA proteins generated in this study and their sizes. (B) Time course of 3D7-Hyp8-HA protein expression from the endogenous locus in *P. falciparum*. Immunoblot of parasite-infected erythrocyte lysates with α-HA and rabbit α-Hyp8 antibodies shows the protein is expressed from 8 h posterythrocyte invasion and protein levels are maintained throughout the parasite lifecycle. Aldolase was used as a loading control. The blot validates that rabbit α-Hyp8 antibodies are specific. (C) Immunofluorescence micrograph of ring-stage parasite-infected erythrocytes probed with α-HA and rabbit α-Hyp8 antibodies shows the protein is exported and localizes to punctate structures (confirmed to be MCs in Figure 6A).
(TIF)

Figure S7 PfEMP1 expression on the erythrocyte surface measured by flow cytometry. (A) Inhibitor treatment regime used to measure PfEMP1 surface display on infected erythrocytes while maintaining parasite viability. Early ring parasites receive inhibitor at 15 µM for 23 h (15 µM), or 50 µM for 12 h followed by a reduction to 15 µM for 11 h (50>15 µM) to maintain parasite viability. (B) Measurement of surface-exposed PfEMP1 on erythrocytes infected with CS2-GFP parasites by flow cytometry using monoclonal human PAM1.4 serum (specific for VAR2CSA). In each plot, the lower left gate corresponds to GFP-negative (uninfected) erythrocytes. The upper left gate corresponds to GFP-positive, surface PfEMP1-negative erythrocytes. The upper right gate corresponds to GFP-positive, surface PfEMP1-positive erythrocytes. Using all GFP-positive cells (upper left and right gates) the geometric mean fluorescence of PAM1.4-labelled cells (channel FL4) was quantified (i.e., the gradient of surface PfEMP1 positivity) from a total input of >100,000 infected and uninfected cells per condition. Parasitemia corresponds to all GFP-positive cells. Plot of uninfected erythrocytes is also shown. Upper panels (“With PAM4.1”) contain cells labeled with primary, secondary, and tertiary antibodies, while lower panels (“Without PAM4.1”) contain cells labeled with secondary and tertiary antibodies only, to show specificity.
(TIF)

Materials and Methods S1 Supplementary Materials and Methods.
(DOCX)

## Abbreviations

DAPI: 4′-6-Diamidino-2-phenylindole
ER: endoplasmic reticulum
GFP: green fluorescent protein
GlcN: glucosamine
HA: hemagglutinin
KAHRP: knob-associated histidine-rich protein
MCs: Maurer's clefts
PEXEL: *Plasmodium* export element
PfEMP1: *P. falciparum* erythrocyte membrane protein 1
PMIX: Plasmepsin IX
PMV: Plasmepsin V
PvPMV: *P. vivax* PMV
SBP1: skeleton binding protein 1
Sta: statine
VTS: vacuolar transport signal
