# Supplementary figures and images for: Inhibition of Plasmepsin V Activity Demonstrates Its Essential Role in Protein Export, PfEMP1 Display, and Survival of Malaria Parasites
Source: PLoS Biol. 2014 Jul 1;12(7):e1001897. doi: 10.1371/journal.pbio.1001897 (PMC4077696; doi:10.1371/journal.pbio.1001897)

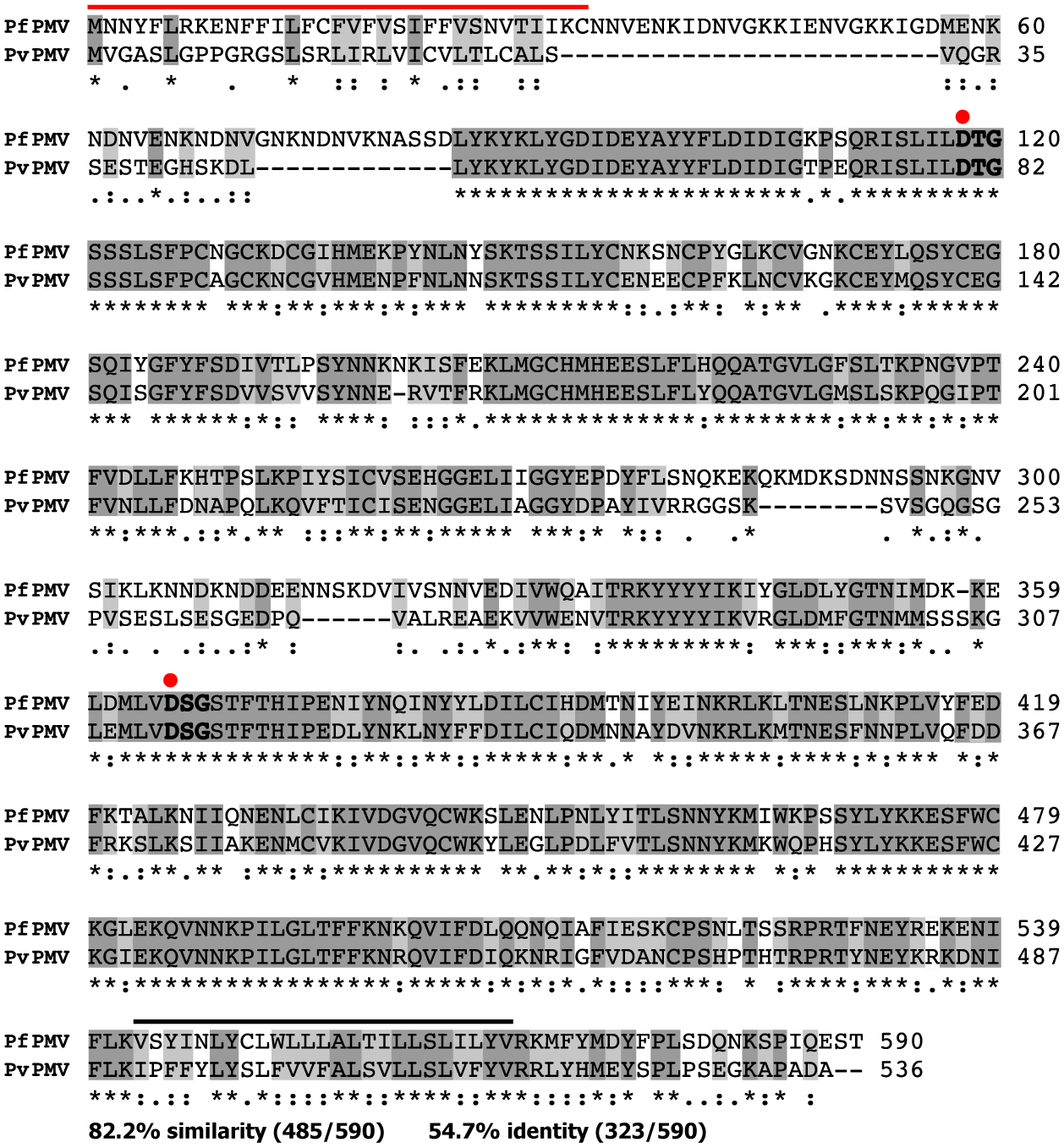

Supplement: Figure S1 — ClustalW alignment of PMV from P. falciparum and P. vivax. The putative P. vivax PMV protein sequence (PvPMV; PVX_116695) was identified by homology searches using the P. falciparum PMV sequence (PfPMV; Pf3D7_1323500). A ClustalW alignment shows they share 82.2% similarity (485/590) and 54.7% identity (323/590) over the full-length sequences including gaps. The predicted signal peptide is shown below the red line, the catalytic dyads are shown in bold with catalytic aspartic acid residues below the red circles, and the C-terminal transmembrane domain is shown below the black line. Four insertions in PfPMV are absent from PvPMV, accounting for their ∼7.5 kDa size difference. (TIF) [file pbio.1001897.s001.tif]

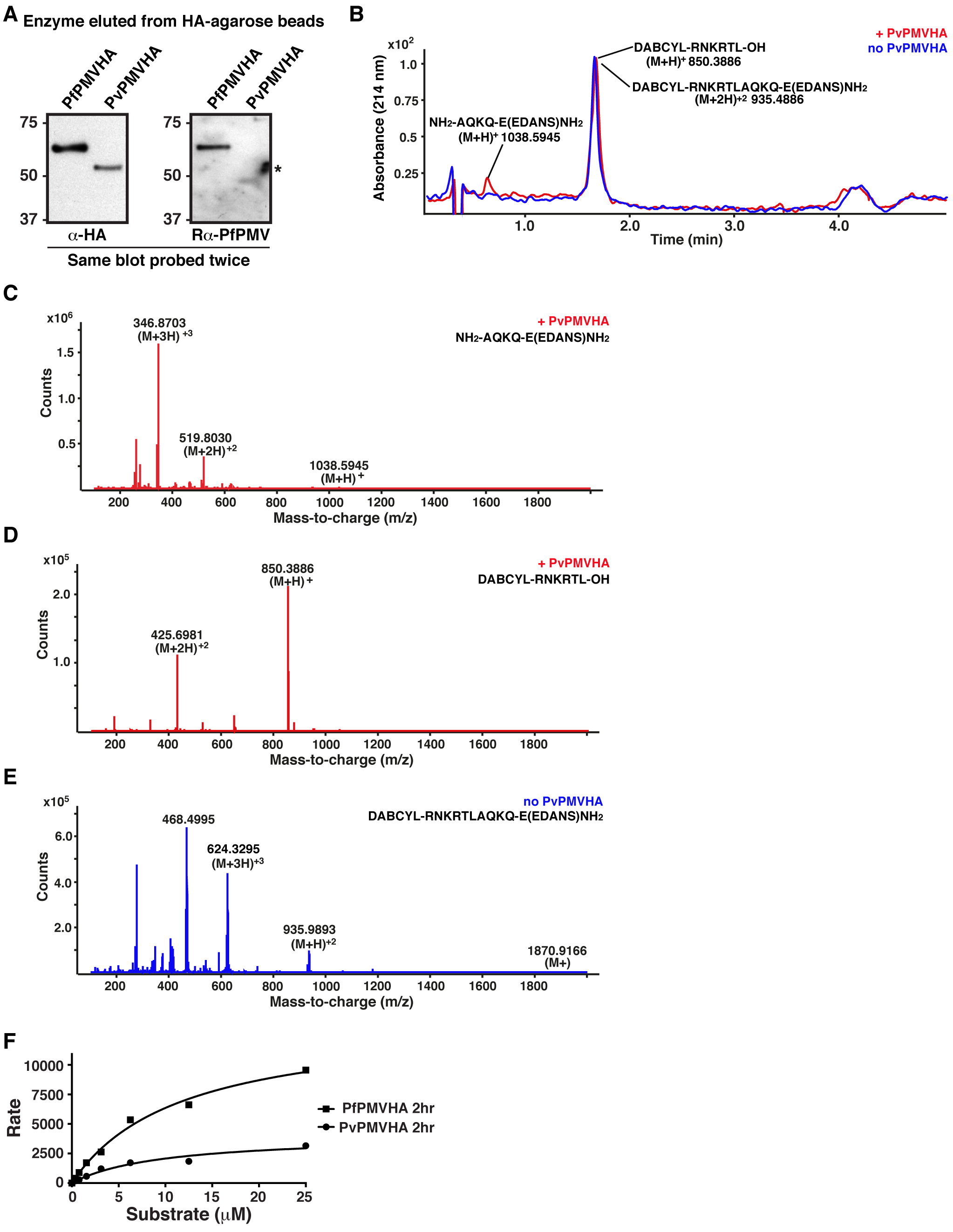

Supplement: Figure S2 — Purity, substrate cleavage position, and kinetics of PMVHA-agarose. (A) Immunoblot of immunopurified PfPMVHA or PvPMVHA eluted from α-HA-agarose with reducing sample buffer shows that the sample preparations contain PMVHA that are species-specific. (Left) α-HA antibodies show purification of PfPMVHA (69.1 kDa once signal peptide removed) and PvPMVHA (60.9 kDa once signal peptide removed) from parasite lysates. (Right) The blot on the left was stripped and reprobed with Rα-PfPMV antibodies. Only HA-tagged PfPMV but not endogenous wild-type PfPMV (64.4 kDa once signal peptide removed) was present in the PfPMV-agarose preparation (lane 1). In lane 2, the Rα-PfPMV antibody does not cross-react with PvPMVHA (60.9 kDa) and does not identify any endogenous wild-type PfPMV in the PvPMVHA-agarose preparation. The black spot on the right of the blot (*) is a nonspecific artefact, not PvPMVHA. (B) LC chromatogram (214 nm) of the fluorogenic KAHRP PEXEL peptide after incubation at 37°C for 48 h with PvPMVHA (red trace) and without PvPMVHA (blue trace). The products of processing by PvPMVHA can be observed: the C-terminal cleavage product NH2-AQKQ-E(EDANS)NH2 (Rf 0.66 min) and the N-terminal fragment DABCYL-RNKRTL-OH (Rf 1.7 min) are labeled. The unprocessed fluorogenic peptide DABCYL-RNKRTLAQKQ-E(EDANS)NH2 is shown to possess an approximately similar Rf (1.7 min) to the processed N-terminal fragment. MS-TOF analysis shows (C) the C-terminal cleavage product (↓AQKQ-E-EDANS), (D) the N-terminal cleavage product (DABCYL-RNKRTL↓), and (E) the unprocessed fluorogenic KAHRP peptide. (F) Michaelis–Menten curve showing the rate of cleavage (relative fluorescence units per min) of increasing concentrations of fluorogenic KAHRP PEXEL peptide by PfPMVHA (squares) and PvPMVHA (circles) after 2 h. The data were used to derive Km values reported in the text. (TIF) [file pbio.1001897.s002.tif]

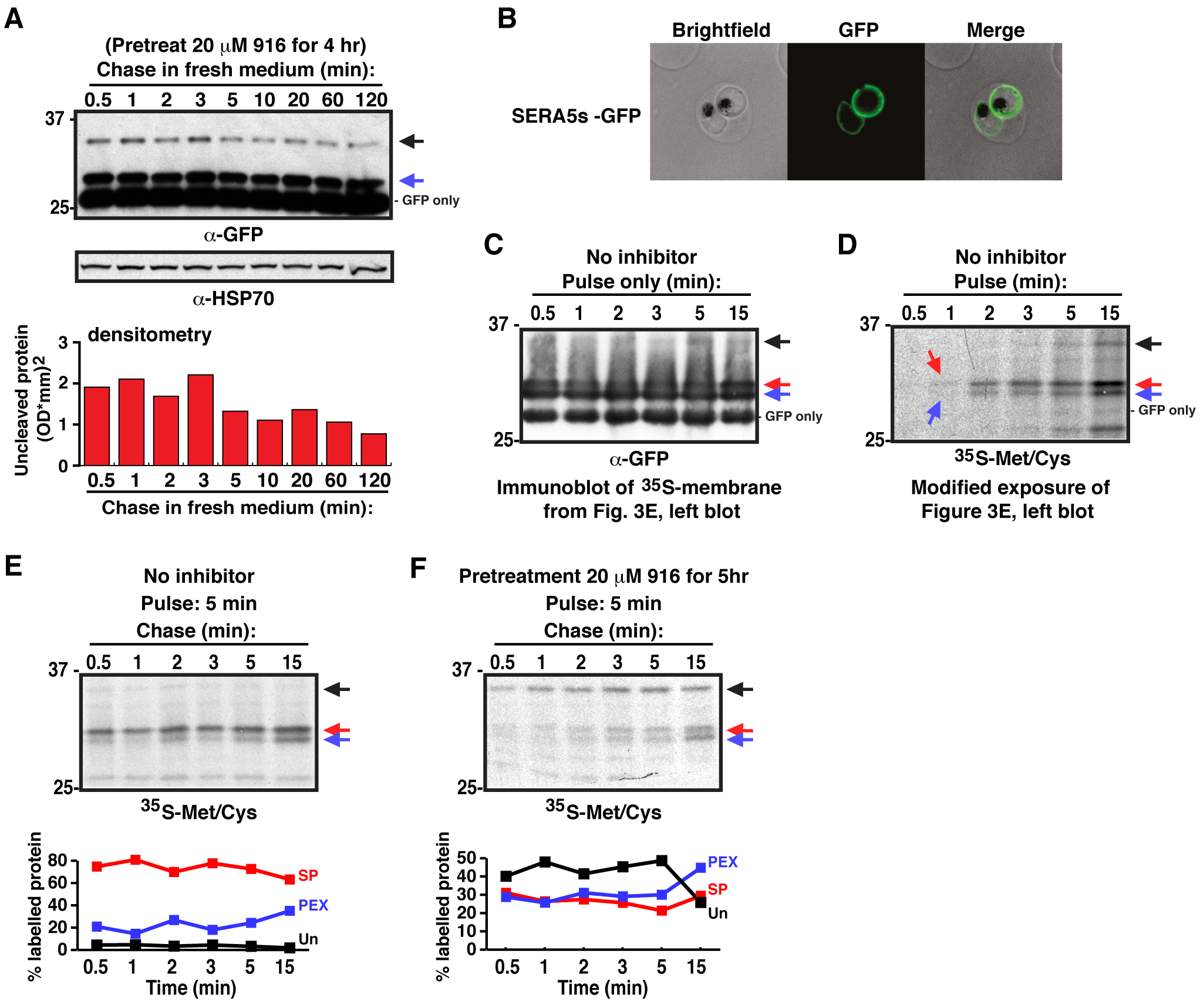

Supplement: Figure S3 — WEHI-916 inhibits PMV in P. falciparum. (A) Immunoblot with anti-GFP antibodies shows that cleavage inhibition of PfEMP3-GFP by 916 treatment (20 µM 916 for 4 h) is rescued after culture in inhibitor-free medium for the indicated times. Densitometry of the uncleaved band is shown below each lane, and HSP70 is a loading control. (B) Immunofluorescence micrograph of P. falciparum expressing SERA5s-GFP, which lacks a PEXEL, showing the chimera is secreted to the parasitophorous vacuole. (C) Immunoblot of the 35S-membrane in Figure 3E with anti-GFP antibodies confirms the uncleaved (black arrow), signal peptide-cleaved (red arrow), PEXEL-cleaved (blue arrow), and GFP-only bands in Figure 3E are indeed GFP-specific. (D) Modified exposure of the blot in Figure 3E, showing the presence of signal peptide-cleaved (red arrow) and PEXEL-cleaved (blue arrow) protein 1 min after addition of label to the culture medium. (E) Pulse chase of PfEMP3-GFP in P. falciparum–infected erythrocytes. (Left) Radiolabeling of PfEMP3-GFP for 5 min (pulse) followed by culture in label-free, inhibitor-free medium for the indicated times (chase) revealed little uncleaved protein (black arrow) and that signal peptide-cleaved protein was more abundant than PEXEL-cleaved protein. The proportion of PEXEL-cleaved protein increased after 3 min of the chase, as the proportion of signal peptide-cleaved protein decreased, demonstrating that PMV can cleave the signal peptide-cleaved protein. (F) Accumulation of uncleaved radiolabeled PfEMP3-GFP in parasites following 5 h of 916 pretreament (20 µM) was reduced after 5–15 min of the chase in label-free, inhibitor-free medium. A concomitant increase in the proportion of PEXEL-cleaved protein was observed from 5 min onward as the quantity of uncleaved protein sharply decreased, demonstrating that PMV can cleave the full-length protein. (TIF) [file pbio.1001897.s003.tif]

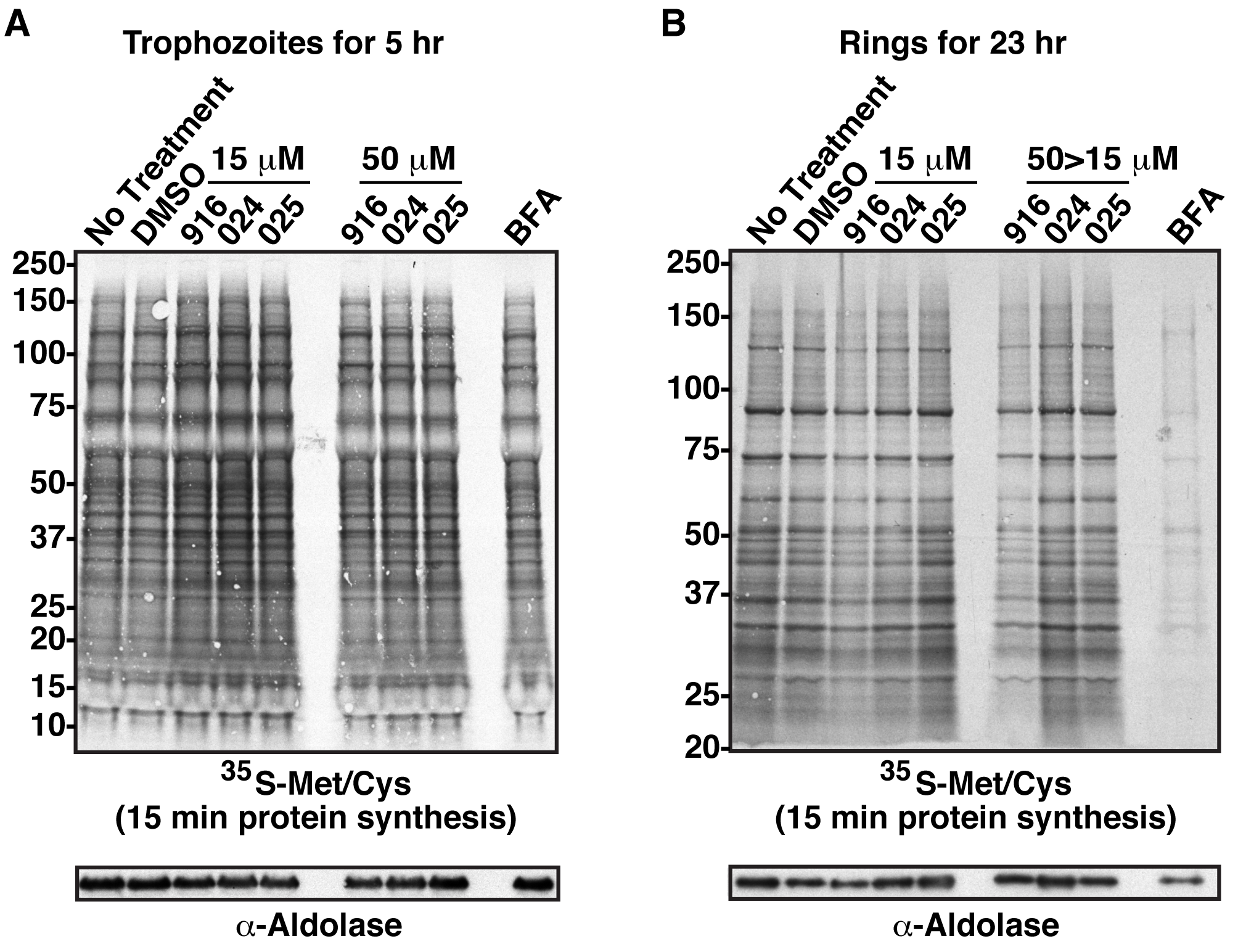

Supplement: Figure S4 — Effect of WEHI-916 on global protein translation in P. falciparum. (A) Magnet-purified trophozoites treated with DMSO or 916, 024, 025 (15 or 50 µM), or Brefeldin A (BFA; 10 µg/ml) for 5 h prior to labeling parasite proteins with 35S-Methionine/Cysteine for 15 min reveal no defect in global protein synthesis. Aldolase levels were examined by immunoblot of the same blot as a loading and viability control. (B) Ring parasites treated with DMSO or 916, 024, 025 (15 µM or 50 µM for 12 h followed by 15 µM for 11 h (50>15 µM)) or BFA (5 µg/ml) for 23 h prior to labeling parasite proteins with 35S-Methionine/Cysteine for 15 min reveals no defect in global protein synthesis for DMSO, 024, or 025. A slight reduction in translation is seen with 916 treatment, and immunoblotting revealed that Aldolase levels were also reduced following 916 treatment, suggesting either that accumulation of uncleaved PEXEL precursors in the ER has a negative effect on translation or that a proportion of parasites were beginning to die or both. BFA treatment, which halts retrograde transport and ER exit, severely reduced translation in parasites and Aldolase levels, suggesting negative feedback on protein synthesis occurs in parasites with blocked ER transport. The effect of 916 was clearly different to that of 024 and 025, suggesting the latter impart toxicity independent of PMV at concentrations >20 µM. (TIF) [file pbio.1001897.s004.tif]

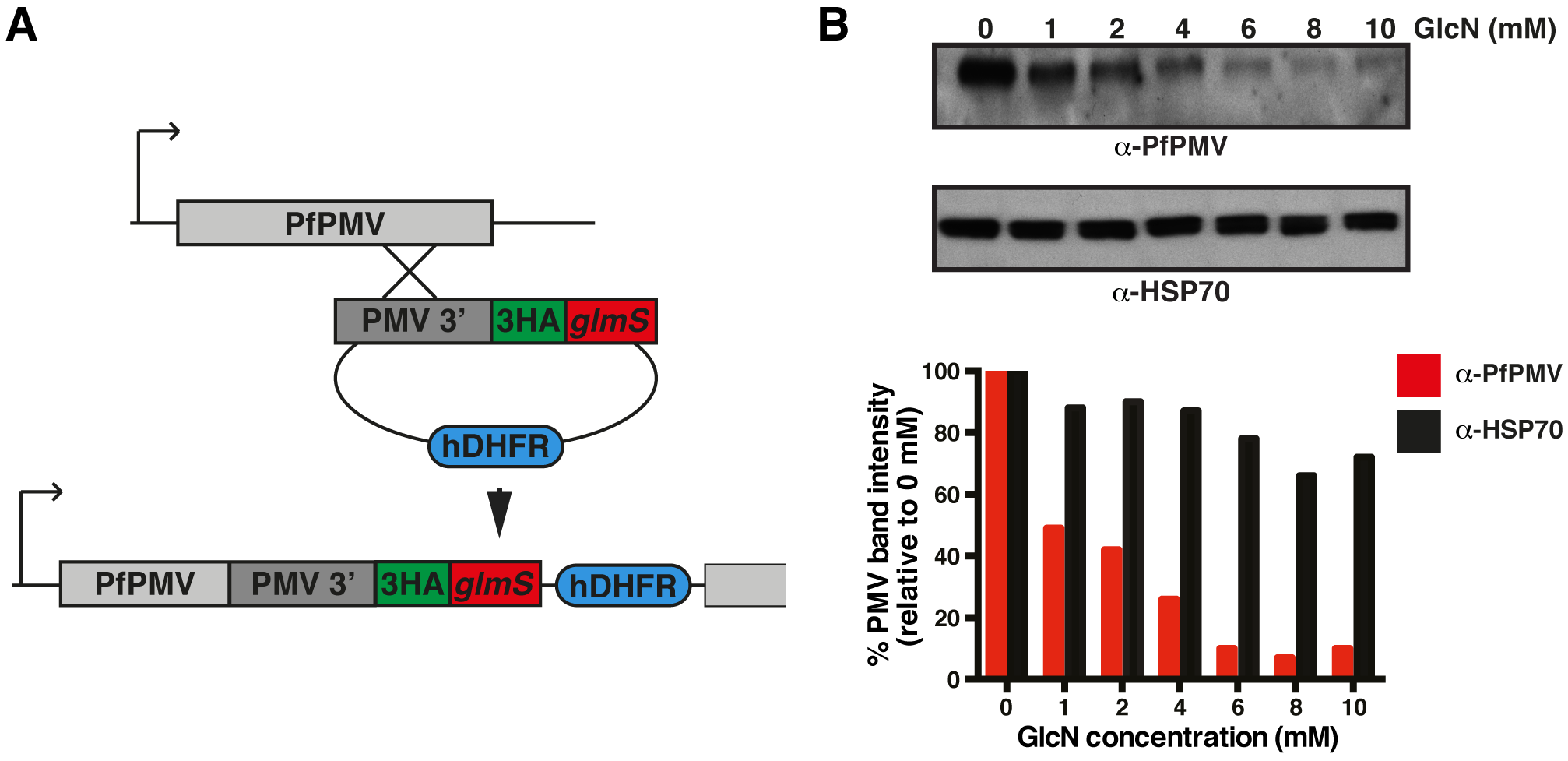

Supplement: Figure S5 — Effect of GlcN addition to P. falciparum cultures. (A) Schematic of allelic exchange to introduce 3× HA epitopes and glmS riboswitch into the 3′ of the PMV gene in P. falciparum NF54. (B) P. falciparum trophozoites were treated with 0–10 mM GlcN for 48 h and PMV levels assessed by immunoblot with α-PfPMV antibodies. HSP70 was used as a loading control and to assess parasite viability in the presence of GlcN. Densitometry of the PMV and HSP70 bands is shown below the blots. We utilized 4–6 mM GlcN for all future experiments. (TIF) [file pbio.1001897.s005.tif]

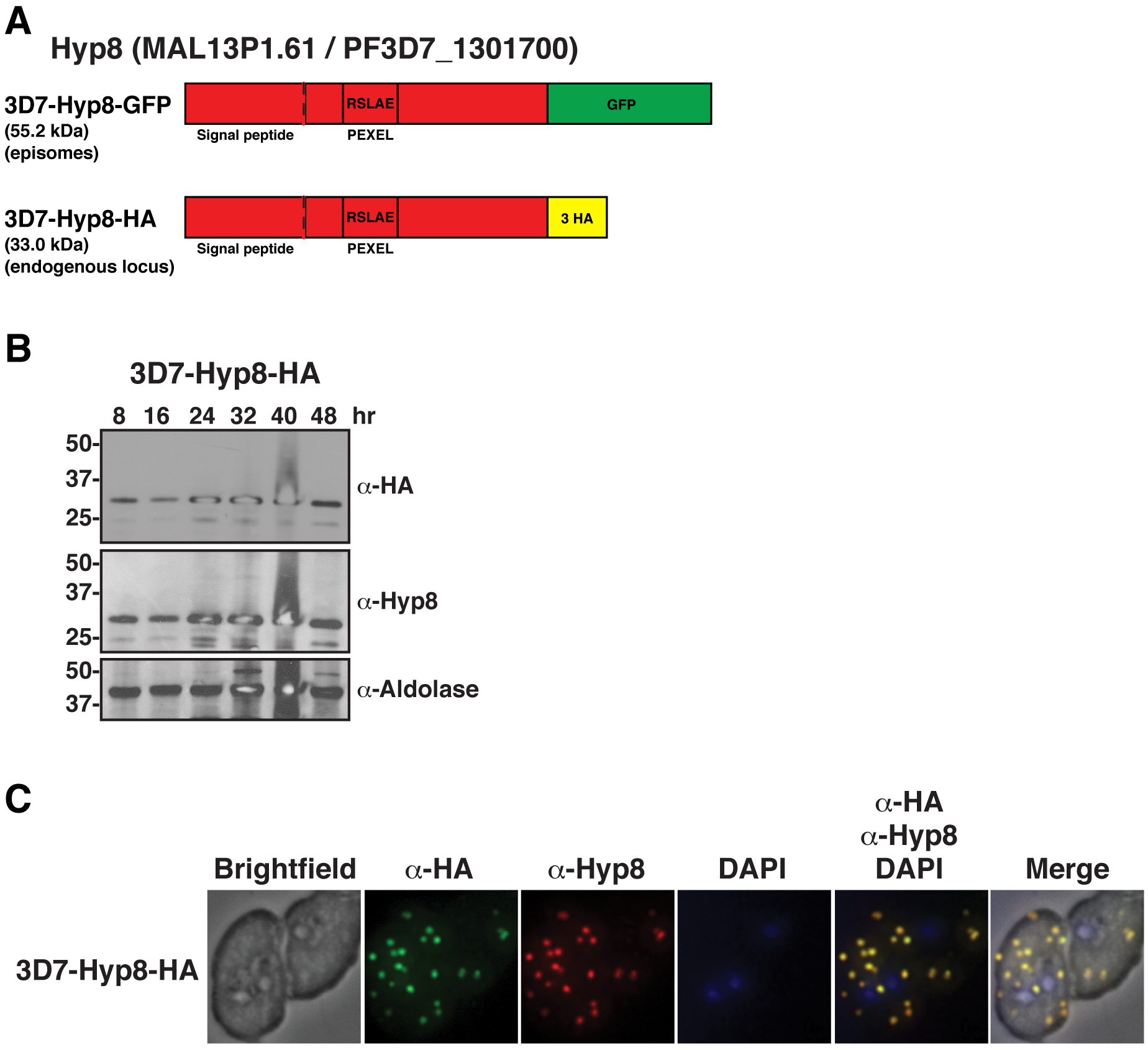

Supplement: Figure S6 — Hyp8 epitope tagging, expression, and export. (A) Schematic of Hyp8-GFP and Hyp8-HA proteins generated in this study and their sizes. (B) Time course of 3D7-Hyp8-HA protein expression from the endogenous locus in P. falciparum. Immunoblot of parasite-infected erythrocyte lysates with α-HA and rabbit α-Hyp8 antibodies shows the protein is expressed from 8 h posterythrocyte invasion and protein levels are maintained throughout the parasite lifecycle. Aldolase was used as a loading control. The blot validates that rabbit α-Hyp8 antibodies are specific. (C) Immunofluorescence micrograph of ring-stage parasite-infected erythrocytes probed with α-HA and rabbit α-Hyp8 antibodies shows the protein is exported and localizes to punctate structures (confirmed to be MCs in Figure 6A). (TIF) [file pbio.1001897.s006.tif]

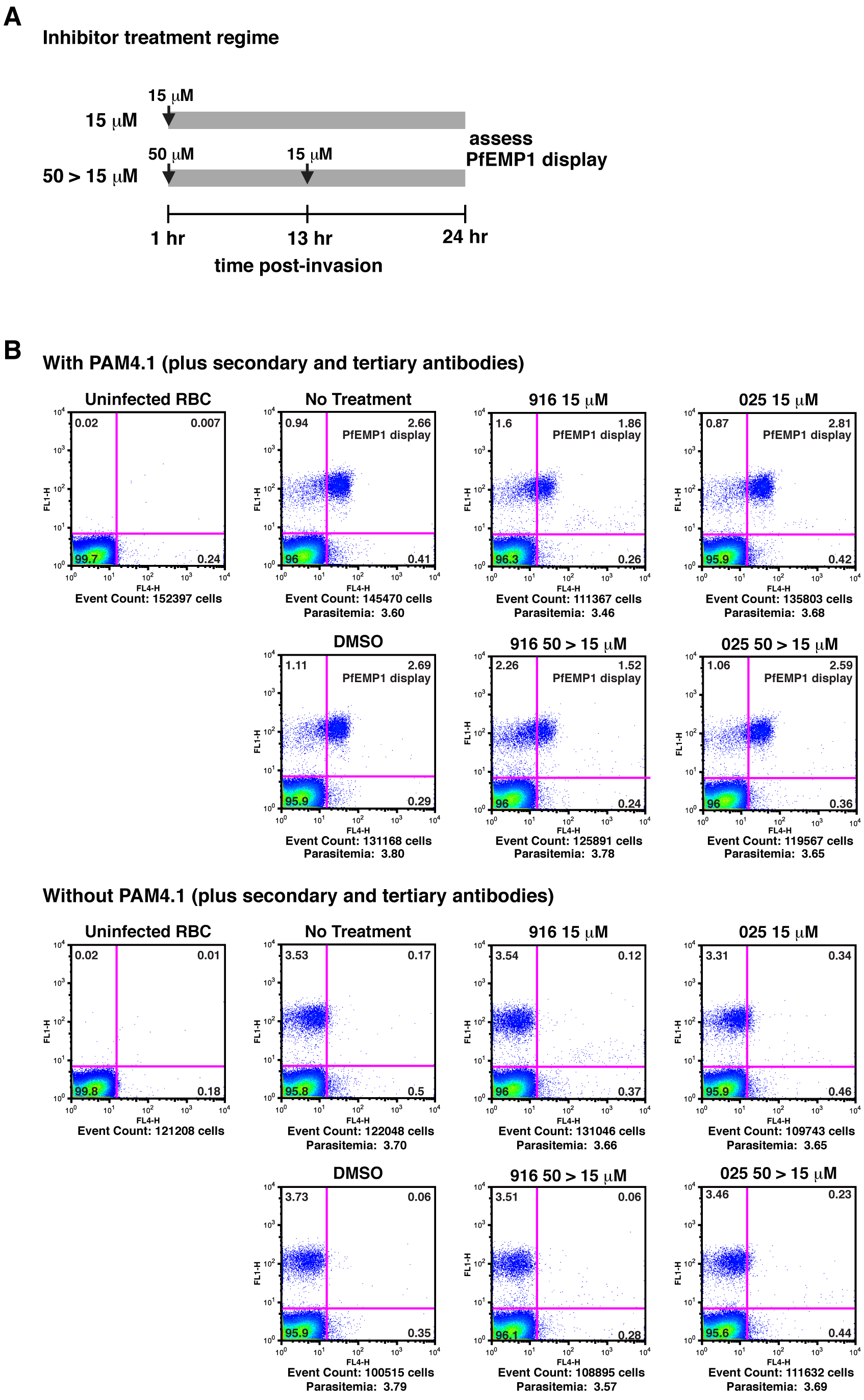

Supplement: Figure S7 — PfEMP1 expression on the erythrocyte surface measured by flow cytometry. (A) Inhibitor treatment regime used to measure PfEMP1 surface display on infected erythrocytes while maintaining parasite viability. Early ring parasites receive inhibitor at 15 µM for 23 h (15 µM), or 50 µM for 12 h followed by a reduction to 15 µM for 11 h (50>15 µM) to maintain parasite viability. (B) Measurement of surface-exposed PfEMP1 on erythrocytes infected with CS2-GFP parasites by flow cytometry using monoclonal human PAM1.4 serum (specific for VAR2CSA). In each plot, the lower left gate corresponds to GFP-negative (uninfected) erythrocytes. The upper left gate corresponds to GFP-positive, surface PfEMP1-negative erythrocytes. The upper right gate corresponds to GFP-positive, surface PfEMP1-positive erythrocytes. Using all GFP-positive cells (upper left and right gates) the geometric mean fluorescence of PAM1.4-labelled cells (channel FL4) was quantified (i.e., the gradient of surface PfEMP1 positivity) from a total input of >100,000 infected and uninfected cells per condition. Parasitemia corresponds to all GFP-positive cells. Plot of uninfected erythrocytes is also shown. Upper panels (“With PAM4.1”) contain cells labeled with primary, secondary, and tertiary antibodies, while lower panels (“Without PAM4.1”) contain cells labeled with secondary and tertiary antibodies only, to show specificity. (TIF) [file pbio.1001897.s007.tif]
