# Supplementary material for: Inhibition of Plasmepsin V Activity Demonstrates Its Essential Role in Protein Export, PfEMP1 Display, and Survival of Malaria Parasites
Source: PLoS Biol. 2014 Jul 1;12(7):e1001897. doi: 10.1371/journal.pbio.1001897 (PMC4077696; doi:10.1371/journal.pbio.1001897)
Supplement: Materials and Methods S1 — Supplementary Materials and Methods. (DOCX) [file pbio.1001897.s008.docx]

**Supporting Information**

**MATERIALS AND METHODS S1**

**PMV modelling.**

Comparative modelling of the structure of PfPMV in complex with 916 was performed using the MODELLER program ([Sali and Blundell, 1993](#_ENREF_9)). The X-ray crystal structures of Plasmepsin II from *P. falciparum* (2BJU) ([Prade et al., 2005](#_ENREF_8)), plasmepsin from *P. vivax* (1QS8) ([Silva et al., 1996](#_ENREF_10)), human BACE-1 (2VIE) ([Clarke et al., 2008](#_ENREF_3)), and secreted aspartic protease (3PVK) ([Behnen et al., 2012](#_ENREF_1)) were used as templates. The resulting model was subjected to 5 ns molecular dynamics (MD) simulation using the GROMACS (v4.5.3) program ([Hess et al., 2008](#_ENREF_6)) employing the OPLS-aa force field([Jorgensen and Tiradorives, 1988](#_ENREF_7)). The system was solvated in a box of water (TIP4P). Ionizable residues were fixed in their charged state, and the system neutralized and the ionic concentration adjusted to 0.1 M by including Na^+^ and Cl^-^ ions. The system was initially minimized prior to MD simulation. Protein and ligand with water and ions were coupled separately to a thermal bath at 300K using velocity rescaling ([Bussi et al., 2007](#_ENREF_2)) applied with a coupling time of 0.1 ps, whereas the pressure was coupled to an isotropic barostat using a time constant of 1 ps and compressibility of 4.5 x 10^-5^ bar^-1^. All simulations were performed with a single non-bonded cutoff of 10 Å and applying a neighbour-list update frequency of 10 steps (20fs). The particle-mesh Ewald method ([Essmann et al., 1995](#_ENREF_4)) was used to account for long-range electrostatics, applying a grid width of 1.2 Å and a fourth-order spline interpolation. Bond lengths were constrained using the LINCS algorithm ([Hess, 2008](#_ENREF_5)).

**General Chemistry Methods**

Analytical thin-layer chromatography was performed on Merck silica gel 60F^254^ aluminum-backed plates and were visualized by fluorescence quenching under UV light or by KMnO_4_ staining. Flash chromatography was performed with silica gel 60 (particle size 0.040-0.063 μm). NMR spectra were recorded on a Bruker Avance DRX 300 with the solvents indicated (^1^H NMR at 300 MHz) (^13^C NMR at 75 MHz). Chemical shifts are reported in ppm on the δ scale and referenced to the appropriate solvent peak. MeOD contains H_2_O. HRESMS were acquired by Jason Dang at the Monash Institute of Pharmaceutical Sciences Spectrometry Facility using an Agilent 1290 infinity 6224 TOF LCMS. Column used was RRHT 2.1 x 50 mm 1.8 µm C18. Gradient was applied over the 5 min with the flow rate of 0.5 mL/min. For MS: Gas temperature was 325^o^C; drying gas 11 L/min; nebulizer 45 psig and the fragmentor 125V. LCMS were recorded on a Waters ZQ 3100 using a 2996 Diode Array Detector. LCMS conditions used to assess purity of compounds were as follows, column: XBridge TM C18 5 µm 4.6 x 100 mm, injection volume 10 µL, gradient: 10-100% B over 10 min (solvent A: water 0.1% formic acid; solvent B: AcCN 0.1% formic acid), flow rate: 1.5 mL/min, detection: 100-600 nm. All final compounds were analyzed using ultrahigh performance liquid chromatography/ultraviolet/evaporative light scattering detection coupled to mass spectrometry. Unless otherwise noted, all compounds were found to be >95% pure by this method.

**Chemistry Experimental**

The following starting materials were purchased commercially and used without further purification, Cbz-Orn(*N*-Boc)-OH, Cbz-Lys(*N*-Boc)-OH .DCHA, Boc-Sta(*S*,*S*)OH, Boc-AHMHpA(3*S*,4*S*,5*S*)-OH .DCHA, and HCl.NH_2_-Val-OMe.

**

**

**Supplementary Scheme 1.** Synthesis of **WEHI-916**.

*Reagents and conditions:* a) HBTU, Et_3_N, DMF, HCl.NH_2_-Val-OMe; b) Pd/C, H_2_, EtOAc; c) PhCH_2_SO_2_Cl, Et_3_N, DCM; d) LiOH.H_2_O, THF, H_2_O; e) HBTU, Et_3_N, DMF, Ph(CH_2_)_2_NH_2_; f) 4N HCl, dioxane; g) HBTU, Et_3_N, DMF, **5**; h) 4N HCl, dioxane; i) Et_3_N, *N*,*N*'-bis-Boc-1-guanylpyrazole; j) TFA, DCM.

**General Procedure A**

**Cbz-Orn(*N*-Boc)-Val-OMe 1**

A mixture of Cbz-Orn(*N*-Boc)-OH (200 mg, 0.546 mmol), Et_3_N (380 μL, 2.73 mmol), HCl.NH_2_-Val-OMe (183 mg, 1.1 mmol), and HBTU (248 mg, 0.665 mmol), in DMF (2.0 mL) was allowed to stir for 18 h at 20^o^C. 10% Citric acid solution was added to the reaction mixture. The solution was extracted with EtOAc (2 x 10 mL). The organic layer was then washed with 10% NaHCO_3_ solution (10 mL). The organic layer was dried (MgSO_4_) and the organic layer was concentrated *in vacuo* to obtain an oil. The oil obtained was subjected to silica chromatography gradient eluting with 100% DCM to 10% MeOH/DCM to obtain **1** as a white solid (225 mg, 86 %). ^1^H NMR (CDCl_3_): δ 7.35 (s, 5H), 6.67 (br s, 1H), 5.45 (br s, 1H), 5.13 (s, 2H), 4.65 (br s, 1H), 4.54-4.50 (m, 1H), 4.37 (br s, 1H), 3.74 (s, 3H), 3.35-3.05 (m, 2H), 2.25-215 (m, 1H), 1.97-1.50 (m, 4H), 1.45 (s, 9H), 0.95-0.91 (m, 6H). MS, *m/z* = 480 [M+H]^+^.

**General Procedure B**

**PhCH_2_SO_2_-Orn(*N*-Boc)-Val-OMe 2**

A mixture of **1** (295 mg, 0.62 mmol) and Pd/C (cat.) in EtOAc (20 mL) under a hydrogen atmosphere was allowed to stir for 18 h. The mixture was filtered through Celite and concentrated to dryness *in vacuo*. To the crude oil was dissolved in DCM (10 mL), benzylsulfonyl chloride (151 mg, 0.79 mmol and Et_3_N (110 μL, 0.79 mmol) was added. The mixture was then allowed to stir for 18 h at 20^o^C. The reaction mixture was concentrated to dryness *in vacuo.* The residue obtained was subjected to silica chromatography gradient eluting with 100% DCM to 5% MeOH/DCM to obtain **2** as a white solid (240 mg, 79%). ^1^H NMR (CDCl_3_): δ 7.44-7.33 (m, 5H), 6.97 (d, 1H, *J* 8.7 Hz), 5.60 (d, 1H, *J* 8.5 Hz), 4.87 (br s, 1H), 4.53-4.48 (m, 1H), 4.25 (s, 2H), 4.06 (br s, 1H), 3.72 (s, 3H), 3.25-2.95 (m, 2H), 2.24-2.15 (m, 1H), 1.84-1.78 (m, 1H), 1.64-1.43 (m, 13H), 0.96 (t, 6H, *J* 6.7 Hz). MS, *m/z* = 500 [M + H]^+^.

**General Procedure C**

**PhCH_2_SO_2_-Orn(*N*-Boc)-Val-OH 3**

A mixture of **2** (180 mg, 0.36 mmol), and LiOH hydrate (24 mg, 0.72 mmol) in a mixture of water (3 mL) and THF (5 mL) was allowed to stir for 2 h at 20^o^C. 10% Citric acid solution was added to the reaction mixture. The solution was extracted with EtOAc (2 x 20 mL). The organic layer was then washed with brine (20 mL). The organic layer was dried (MgSO_4_) and the organic layer was concentrated *in vacuo* to obtain **3** as a colourless oil (170 mg, 97%). ^1^H NMR (CDCl_3_) (rotamers): δ 7.43-7.36 (5H, m), 7.09 (br s, 1H), 5.90-5.75 (m, 1.5H), 4.91 (br s, 0.5H), 4.49 (br s, 1H), 4.26 (s, 2H), 4.02-3.94 (m, 1H), 3.20-2.95 (m, 2H), 2.31-2.20 (m, 1H), 1.89-1.40 (13H, m), 1.02-0.98 (m, 6H). MS, *m/z* = 486 [M + H]^+^.

**Boc-Sta-NH(CH_2_)_2_Ph 4**

General Procedure A was followed using Boc-Sta(S,S)-OH (500 mg, 1.82 mmol), and phenethylamine (468 mg, 3.63 mmol) to obtain **4** as an oil (680 mg, 98%). ^1^H NMR (CDCl_3_): δ 7.32-7.19 (m, 5H), 6.34 (brs, 1H), 4.82 (d, 1H, *J* 9.6 Hz), 3.95-3.90 (m, 1H), 3.56-3.48 (m, 3H), 2.85-2.75 (m, 2H), 2.40-2.23 (m, 2H), 1.67-1.20 (m, 12H), 0.93-0.90 (m, 6H). MS, *m/z* = 379 [M + H]^+^.

**General Procedure D**

**NH_2_-Sta-NH(CH_2_)_2_Ph. HCl 5**

A mixture of **4** (680 mg, 1.8 mmol), in a mixture of 4N HCl in dioxane (4.0 mL) was allowed to sit for 2 h at 20^o^C. The reaction mixture was concentrated to dryness *in vacuo* to obtain a solid. The solid was triturated with Et_2_O and filtered off to obtain **5** as a white solid (550 mg, 97%). ^1^H NMR (MeOD): δ 7.35-7.18 (m, 5H), 4.03-3.97 (m, 1H), 3.44 (t, 2H, *J* 7.1 Hz), 3.34-3.22 (m, 1H), 2.85-2.80 (m, 2H), 2.58-2.42 (m, 2H), 1.80-1.70 (m, 1H), 1.63-1.44 (m, 2H), 1.01-0.94 (m, 6H). MS, *m/z* = 279 [M + H]^+^.

**PhCH_2_SO_2_-Orn(*N*-Boc)-Val-Sta-NH(CH_2_)_2_Ph 6**

General Procedure A was followed using **3** (100 mg, 0.21 mmol), and **5** (84 mg, 0.27 mmol), to obtain **6** as a white solid (110 mg, 72%). ^1^H NMR (CDCl_3_) (rotamers): δ 7.63 (br s, 1H), 7.45-7.10 (m, 10H), 6.87 (br s, 1H), 6.29 (br s, 1H), 5.00 (br s, 1H), 4.35-4.20 (m, 3H), 4.05-3.80 (m, 3H), 3.50-3.30 (m, 2H), 3.15-3.00 (m, 2H), 2.81-2.69 (m, 2H), 2.35-2.25 (m, 2H), 2.15-2.02 (m, 1H), 1.70-1.30 (m, 16H), 0.99-0.85 (m, 12H). MS, *m/z* = 746 [M + H]^+^.

**General Procedure E**

**PhCH_2_SO_2_-Arg(*N,N*-diBoc)-Val-Sta-NH(CH_2_)_2_Ph 7**

A mixture of **6** (55 mg, 0.07 mmol), in 4N HCl in dioxane (2 mL) was allowed to stir for 30 min at 20^o^C. The reaction mixture was concentrated to dryness *in vacuo*. The residue was dissolved in dichlormethane (10 mL) and Et_3_N (51 μL, 0.370 mmol) was added. The solution was stirred vigorously for 5 min. *N*,*N*'-bis-Boc-1-guanylpyrazole (28 mg, 0.088 mmol) was added and the solution was left to stir for 12 h. 10% Citric acid solution was added to the reaction mixture. The solution was extracted with DCM (2 x 15 mL). The organic layer was then washed with 10% NaHCO_3_ solution (20 mL). The organic layer was dried (MgSO_4_) and the organic layer was concentrated *in vacuo* to obtain an oil. The oil was subjected to silica chromatography gradient eluting with 100% DCM to 10% MeOH/DCM to obtain **7** as a colourless oil (55 mg, 84%). ^1^H NMR (CDCl_3_) (rotamers): δ 8.85-8.35 (m, 1H), 7.45-7.15 (m, 10H), 7.05 (br s, 0.5H), 6.75-6.55 (m, 1.5H), 6.29 (br s, 0.5H), 5.83 (br s, 0.5H), 4.32-4.22 (m, 4H), 4.05-3.70 (m, 3H), 3.55-3.30 (m, 4H), 2.83-2.72 (m, 2H), 2.35-2.05 (m, 3H), 1.80-1.30 (25H, m), 0.98-0.84 (m, 12H). MS, *m/z* = 888 [M]^+^.

**General Procedure F**

**PhCH_2_SO_2_-Arg(NH_2_)-Val-Sta-NH(CH_2_)_2_Ph .TFA (WEHI-916)**

A mixture of **7** (40 mg, 0.05 mmol), in TFA (0.5 mL) and DCM (1 mL) was allowed to sit for 18 h at 20^o^C. The reaction mixture was concentrated to dryness *in vacuo*. The oil was triturated with Et_2_O and filtered off, washing with Et_2_O, to obtain **WEHI-916** as a white solid (35 mg, 97%). ^1^H NMR (MeOD): δ 7.46-7.18 (m, 10H), 4.33 (s, 2H), 4.19-4.14 (m, 1H), 4.00-3.91 (m, 3H), 3.44-3.38 (m, 1H), 3.22-3.16 (m, 2H), 2.83-2.72 (m, 2H), 2.27-2.23 (m, 2H), 2.17-2.05 (m, 1H), 1.80-1.50 (m, 6H), 1.37-1.31 (m, 1H), 1.05-0.86 (m, 12H). ^13^C NMR (MeOD) (rotamers): δ 174.29, 173.99, 173.50, 173.45, 158.63, 158.61, 140.53, 140.51, 132.13, 132.06, 131.09, 130.88, 130.84, 129.78, 129.76, 129.74, 129.67, 129.65, 129.61, 129.59, 129.55, 129.52, 129.49, 129.47, 129.35, 127.32, 71.58, 71.34, 60.90, 60.76, 60.22, 60.11, 57.76. 57.79, 53.02, 52.78, 42.09, 42.08, 41.90, 41.69, 41.52, 41.08, 40.91, 36.53, 36.50, 31.59, 31.57, 26.27, 26.03, 25.93, 25.81, 23.75, 23.44, 22.24, 22.04, 20.05, 19.88, 18.71, 18.68. HRESMS found: (M + H) 688.3850; C_34_H_53_N_7_O_6_S requires (M + H), 688.3856.

**

**

**Supplementary Scheme 2.** Synthesis of **WEHI-025**.

*Reagents and conditions:* a) HBTU, Et_3_N, DMF, Ph(CH_2_)_2_NH_2_; b) 4N HCl, dioxane; c) HBTU, Et_3_N, DMF, **9**; d) 4N HCl, dioxane; e) Et_3_N, *N*,*N*'-bis-Boc-1-guanylpyrazole; f) TFA, DCM.

**Boc-** **AHMHpA-NH(CH_2_)_2_Ph 8**

Boc-AHMHpA(3S,4S,5S)-OH·DCHA (500 mg, 1.82 mmol) was desalted using 10% citric acid solution. The crude material was subjected to General Procedure A using phenethylamine (468 mg, 3.63 mmol) to obtain **8** as an oil (680 mg, 98%). ^1^H NMR (CDCl_3_): δ 7.34-7.19 (m, 5H), 6.37 (br s, 1H), 4.95 (d, 1H, *J* 10 Hz), 4.22-4.18 (m, 1H), 3.56-3.48 (m, 1H), 3.18-3.11 (m, 1H), 2.88-2.85 (m, 2H), 2.41-2.18 (m, 2H), 1.67-1.53 (m, 2H), 1.44 (s, 9H), 1.23-1.10 (m, 1H), 0.95-0.85 (m, 6H). MS, *m/z* = 379 [M+H]^+^.

**NH_2_- AHMHpA-NH(CH_2_)_2_Ph .HCl 9**

General Procedure D was followed using **8** (80 mg, 0.21 mmol), to obtain **9** as an oil (65 mg, 98%). ^1^H NMR (MeOD): δ 7.30-7.20 (m, 5H), 4.23-4.18 (m, 1H), 3.77-3.58 (m, 1H), 3.45 (m, 2H, *J* 7.0 Hz), 3.05-3.02 (m, 1H), 2.83 (t, 1H, *J* 7.6 Hz), 2.60-2.43 (m, 2H), 1.80-1.73 (m, 1H), 1.60-1.52 (m, 1H), 1.27-1.15 (m, 1H), 1.05 (d, 3H, *J* 6.9 Hz), 0.98 (t, 2H, *J* 7.3 Hz). MS, *m/z* = 279 [M+H]^+^.

**PhCH_2_SO_2_Orn(Boc)-Val-AHMHpA-NH(CH_2_)_2_Ph 10**

General Procedure A was followed using **9** (80 mg, 0.165 mmol) and **3** (62 mg, 0.19 mmol) to obtain an oil. The oil was subjected to silica chromatography gradient eluting with 100% DCM to 10% MeOH/DCM to obtain **10** as a white solid (75 mg, 61%). ^1^H NMR (CDCl_3_): δ 7.41-6.95 (m, 10H), 6.95 (br s, 1H), 6.65 (br s, 1H), 6.03 (br s, 1H), 4.87 (br s, 1H), 4.27-4.23 (3H, m), 3.94-3.80 (1H, m), 3.60-3.40 (m, 3H), 3.35-3.36 (m, 1H), 3.15-3.00 (m, 2H), 2.83-2.71 (m, 2H), 2.38-2.09 (m, 3H), 1.80-1.45 (m, 15H), 1.20-1.05 (m, 1H), 1.00-0.78 (m, 12H). MS, *m/z* = 746 [M+H]^+^.

**PhCH_2_SO_2_Arg(*N,N*-diBoc)-Val-AHMHpA-NH(CH_2_)_2_Ph 11**

General Procedure E was followed using **10** (40 mg, 0.05 mmol), to obtain **11** as a colourless glass (60 mg, 84%). ^1^H NMR (CDCl_3_): δ 8.65-8.50 (m, 1H), 7.41-7.16 (m, 10H), 6.85-6.70 (m, 1H), 6.60 (br s, 1H), 6.17 and 5.80 (2 x br s, 1H), 4.31-4.23 (m, 4H), 4.90-3.75 (m, 1H), 3.60-3.25 (m, 5H), 2.84-2.72 (m, 2H), 2.40-2.10 (m, 3H), 1.80-1.40 (m, 24H), 1.15-1.05 (m, 1H), 1.02-0.79 (m, 12H). MS, *m/z* = 889 [M+H]^+^.

**PhCH_2_SO_2_Arg-Val-AHMHpA-NH(CH_2_)_2_Ph . TFA (WEHI-025)**

General Procedure F was followed using **11** (50 mg, 0.056 mmol), to obtain **WEHI-025** as a white solid (43 mg, 95%). ^1^H NMR (MeOD): δ 7.44-7.19 (m, 10H), 4.39-3.90 (m, 5H), 3.56-3.16 (m, 5H), 2.84-2.68 (m, 2H), 2.57-2.48 (m, 1H), 2.26-2.13 (m, 2H), 1.80-1.45 (m, 6H), 1.25-1.15 (m, 1H), 1.07-0.85 (m, 12H). ^13^C NMR (MeOD) (rotamers): δ 174.30, 174.04, 173.94, 173.75, 173.61, 158.63, 158.61, 140.54, 140.51, 132.14, 132.05, 129.81, 129.79, 129.77, 129.74, 129.65, 129.61, 129.58, 129.56, 129.53, 129.51, 129.50, 129.46, 129.46, 127.35, 127.30, 68.81, 68.59, 60.95, 60.67, 60.24, 60.17, 59.04, 58.70, 57.94, 57.73, 49.27, 49.01, 42.74, 42.54, 42.02, 41.91, 36.67, 26.49, 36.45, 26.22, 31.66, 31.61, 31.57, 31.48, 31.38, 26.76, 26.65, 26.33, 26.10, 20.18, 20.15, 20.03, 19.99, 18.67, 18.64, 18.55, 18.53. HRESMS found: (M + H) 688.3847; C_34_H_53_N_7_O_6_S requires (M + H), 688.3856.

**

**

**Supplementary Scheme 3.** Synthesis of **WEHI-024**.

*Reagents and conditions:* a) HBTU, Et_3_N, DMF, HCl.NH_2_-Val-OMe; b) Pd/C, H_2_, EtOAc; c) PhCH_2_SO_2_Cl, Et_3_N, DCM; d) LiOH.H_2_O, THF, H_2_O; e) HBTU, Et_3_N, DMF, **5**; f) TFA, DCM.

**Cbz-Lys(*N*-Boc)-Val-OMe 12**

Cbz-Lys(*N*-Boc)-OH .DCHA (750 mg,1.34 mmol) was desalted using 10% citric acid solution. The crude material was subjected to General Procedure A, using HCl. NH_2_-Val-OMe (269 mg, 1.6 mmol) to obtain **12** as a white solid (605 mg, 92%). ^1^H NMR (CDCl_3_): δ 7.36 (s, 5H), 6.51 (br s, 1H), 5.48 (br s, 1H), 5.12 (s, 2H), 4.67 (br s, 1H), 4.55-4.50 (m, 1H), 4.23-4.18 (m, 1H), 3.75 (s, 3H), 3.14-3.08 (m, 2H), 2.22-2.15 (m, 1H), 1.91-1.73 (m, 1H), 1.70-1.40 (m, 14H), 0.94-0.89 (m, 6H). MS, *m/z* = 494 [M+H]^+^.

**PhCH_2_SO_2_-Lys(*N*-Boc)-Val-OMe 13**

General Procedure B was followed using **12** (0.5 g, 1.01 mmol) and benzyl sulfonyl chloride (251 mg, 1.32 mmol), to obtain **13** as a white solid (430 mg, 83%). ^1^H NMR (CDCl_3_): δ 7.46-7.36 (m, 5H), 6.59 (d, 1H, *J* 7.9 Hz), 5.21 (d, 1H, *J* 7.3 Hz), 4.69 (br s, 1H), 4.56-4.51 (m, 1H), 4.27 (s, 2H), 3.85-3.77 (m, 1H), 3.76 (s, 3H), 3.20-3.01 (m, 2H), 2.26-2.18 (m, 1H), 1.82-1.28 (15H), 1.00-0.94 (m, 6H). MS, *m/z* = 514 [M+H]^+^.

**PhCH_2_SO_2_-Lys(*N*-Boc)-Val-OH 14**

General Procedure C was followed using **13** (350 mg, 0.68 mmol), to obtain **14** as a colourless oil (330 mg, 97%). ^1^H NMR (CDCl_3_): δ 7.43-7.37 (m, 5H), 6.86-6.69 (m, 1H), 5.68 (br s, 1H), 4.78 (br s, 1H), 4.53-4.49 (m, 1H), 4.27 (s, 2H), 3.89-3.83 (m, 1H), 3.15-3.05 (m, 2H), 2.28-2.22 (m, 1H), 1.92-1.25 (15H), 1.03-0.96 (m, 6H). MS, *m/z* = 500 [M+H]^+^.

**PhCH_2_SO_2_-Lys(*N*-Boc)-Val-Sta-NH(CH_2_)_2_Ph 15**

General Procedure A was followed using **14** (100 mg, 0.200 mmol) and **5** (82 mg, 0.26 mmol) to obtain **15** a colourless oil (60 mg, 40%). ^1^H NMR (CDCl_3_): δ 7.41-7.15 (10H, m), 6.84 (br s, 2H), 6.27 and 5.96 (2 x br s, 1H), 4.75 (br s, 1H), 4.31-4.23 (m, 3H), 4.02-3.85 (m, 2H), 3.71 (br s, 1H), 3.54-3.29 (m, 3H), 3.06 (br s, 2H), 2.84-2.72 (m, 2H), 2.32-2.10 (m, 3H), 1.70-1.25 (18H), 0.99-0.95 (m, 12H). MS, *m/z* = 761 [M+H]^+^.

**PhCH_2_SO_2_-Lys(NH_2_)-Val-Sta-NH(CH_2_)_2_Ph .TFA (WEHI-024)**

General Procedure F was followed using **15** (40 mg, 0.053 mmol) to obtain **WEHI-024** as a white solid (35 mg, 86%). ^1^H NMR (MeOD): δ 7.43-7.19 (m, 10H), 4.44 (s, 2H), 4.21-4.12 (m, 1H), 4.00-3.91 (m, 3H), 3.44-3.37 (m, 1H), 2.94-2.89 (m, 2H), 2.83-2.70 (m, 2H), 2.27-2.11 (m, 3H), 1.76-1.31 (m, 9H), 1.05-0.87 (m, 12H). ^13^C NMR (MeOD) (rotamers): δ 174.45, 174.20, 173.97, 173.96, 173.50, 173.44, 140.54, 140.53, 132.14, 132.06, 130.93, 130.88, 129.79, 129.77, 129.60, 129.58, 129.55, 129.53, 129.51, 129.49, 129.48, 128.91, 127.35, 127.32, 71.64, 71.28, 60.85, 60.80, 60.23, 60.13, 58.13, 58.02, 52.98, 52.79, 42.08, 41.78, 41.52, 41.06, 41.02, 40.51, 40.50, 36.56, 36.53, 36.50, 33.82, 33.65, 31.57, 28.12, 28.05, 25.93, 25.81, 23.76, 23.75, 23.63, 23.59, 22.34, 22.28, 22.06, 20.05, 19.87. HRESMS found: (M + H) 660.3799; C_34_H_53_N_5_O_6_S requires (M + H), 660.3795.

**Abbreviations**

AHMHpA (3S,4S,5S)-4-amino-3-hydroxy-5-methylheptanoic acid

HBTU 2-(1*H*-benzotriazole-1-yl)-1,1,3,3-tetramethyluronium hexafluorophosphate

Sta Statine

**References**

Behnen, J., Koster, H., Neudert, G., Craan, T., Heine, A., and Klebe, G. (2012). Experimental and computational active site mapping as a starting point to fragment-based lead discovery. ChemMedChem *7*, 248-261.

Bussi, G., Donadio, D., and Parrinello, M. (2007). Canonical sampling through velocity rescaling. The Journal of chemical physics *126*, 014101.

Clarke, B., Demont, E., Dingwall, C., Dunsdon, R., Faller, A., Hawkins, J., Hussain, I., MacPherson, D., Maile, G., Matico, R.*, et al.* (2008). BACE-1 inhibitors part 2: identification of hydroxy ethylamines (HEAs) with reduced peptidic character. Bioorganic & medicinal chemistry letters *18*, 1017-1021.

Essmann, U., Perera, L., Berkowitz, M.L., Darden, T., Lee, H., and Pedersen, L.G. (1995). A smooth particle mesh Ewald method. Journal of Chemical Physics *103*, 8577-8593.

Hess, B. (2008). P-LINCS: A parallel linear constraint solver for molecular simulation. J Chem Theory Comput *4*, 116-122.

Hess, B., Kutzner, C., van der Spoel, D., and Lindahl, E. (2008). GROMACS 4: Algorithms for highly efficient, load-balanced, and scalable molecular simulation. J Chem Theory Comput *4*, 435-447.

Jorgensen, W.L., and Tiradorives, J. (1988). The Opls Potential Functions for Proteins - Energy Minimizations for Crystals of Cyclic-Peptides and Crambin. J Am Chem Soc *110*, 1657-1666.

Prade, L., Jones, A.F., Boss, C., Richard-Bildstein, S., Meyer, S., Binkert, C., and Bur, D. (2005). X-ray structure of plasmepsin II complexed with a potent achiral inhibitor. J Biol Chem *280*, 23837-23843.

Sali, A., and Blundell, T.L. (1993). Comparative protein modelling by satisfaction of spatial restraints. J Mol Biol *234*, 779-815.

Silva, A.M., Lee, A.Y., Gulnik, S.V., Maier, P., Collins, J., Bhat, T.N., Collins, P.J., Cachau, R.E., Luker, K.E., Gluzman, I.Y.*, et al.* (1996). Structure and inhibition of plasmepsin II, a hemoglobin-degrading enzyme from *Plasmodium falciparum*. Proc Natl Acad Sci U S A *93*, 10034-10039.
